# Revealing the complex genetic structure of cultivated amaryllis (*Hippeastrum hybridum*) using transcriptome-derived microsatellite markers

**DOI:** 10.1038/s41598-018-28809-9

**Published:** 2018-07-13

**Authors:** Yi Wang, Defeng Chen, Xiaofeng He, Jiangxian Shen, Min Xiong, Xian Wang, Di Zhou, Zunzheng Wei

**Affiliations:** 1 Beijing Vegetable Research Center, Beijing Academy of Agriculture and Forestry Sciences, Key Laboratory of Biology and Genetic Improvement of Horticultural Crops, Ministry of Agriculture, Key Laboratory of Urban Agriculture, Ministry of Agriculture, Beijing Engineering Technology Research Center of Functional Floriculture, Beijing, 100097 China; 2 Department of Ornamental Horticulture, China Agricultural University, Beijing, 100193 China; 3 Key Laboratory of Genetics and Breeding in Forest Trees and Ornamental Plants, Ministry of Education, College of Biological Sciences and Technology, Beijing Forestry University, Beijing, 100083 China; 4 School of Basic Medical Science, Guiyang Medical University, Guizhou, 550004 China

**Keywords:** Genetic variation, Plant breeding, Plant molecular biology

## Abstract

Although amaryllis (*Hippeastrum hybridum*) plants are commonly used in physiological and ecological research, the extent of their genomic and genetic resources remains limited. The development of molecular markers is therefore of great importance to accelerate genetic improvements in *Hippeastrum* species. In this study, a total of 269 unique genes were defined that might regulate the flower spathe development of amaryllis. In addition, 2000 simple sequence repeats (SSRs) were detected based on 171,462 *de novo* assembled unigenes from transcriptome data, and 66,4091 single nucleotide polymorphisms (SNPs) were also detected as putative molecular markers. Twenty-one SSR markers were screened to evaluate the genetic diversity and population structure of 104 amaryllis accessions. A total of 98 SSR loci were amplified for all accessions. The results reveal that Nei’s gene diversity (H) values of these markers ranged between 0.055 and 0.394, whereas the average values of Shannon’s Information index (I) ranged between 0.172 and 0.567. Genetic tree analysis further demonstrates that all accessions can be grouped into three main clusters, which can be further divided into two subgroups. STRUCTURE-based analysis revealed that the highest ΔK values were observed when K = 5, K = 6, K = 7 and K = 8. The results of this study enable large-scale transcriptomics and classification of *Hippeastrum* genetic polymorphisms and will be useful in the future for resource conservation and production.

## Introduction

The genus Hippeastrum in the family Amaryllidaceae includes 75 species from South America, except for one from the West African taxa^1^. *Hippeastrum*, which is also commonly known as the giant amaryllis, is a bulbous perennial plant that is characterized by a ploidy level that ranges between diploid and octoploid^2^. These plants typically produce between three and six glossy strap-like leaves, which are approximately 600 mm long and 50 mm wide^1,3^; flowers appear simultaneously with the leaves in this genus, and one plant can produce one or two inflorescences. Each of these clusters contains between two and five (mostly four) large trumpet-shaped flowers, which have a scape that is typically hollow and can grow up to 550 mm in size^3,4^. Flowers vary from pure white to brilliant red. The flowers are zygomorphic and approximately 200 mm in diameter, exhibiting numerous variations in colour and striping. Amaryllis seeds are flat and have black, papery wings. The plant is well adapted to a warm and humid environment. In some parts of the world, such as Bangladesh, agro-ecological conditions are extremely conducive to the survival and culture of amaryllis; thus, this plant exhibits significant local economic potential, including the export market^5^. Amaryllis also contain alkaloids; these plants are able to synthesize eight typical skeletal forms of isoquinoline alkaloids that exhibit a wide range of demonstrated biological functions, including antitumoural, antiviral, antiparasitic, and acetylcholinesterase inhibition properties^4^. These plants therefore also have economic benefits for the pharmaceutical industry given their high alkaloid content^6^.

Amaryllis have attracted worldwide attention given their large size, variety of brightly coloured and long-lasting flowers^7^, and greater than 300 cultivars are currently recognized^5^. Varieties of *Hippeastrum* are exported by the Netherlands, South Africa, Japan, Brazil, and the United States^8^ and can be traced back to a breeding plan initiated in 1799^9^ that was based on a simple F_1_ hybrid between *Hippeastrum*. *ambiguum* Herb. ex Hook. and *Hippeastrum*. *papilio* (Rav.) Van Scheepen. Wild diploid species were subsequently crossed with older, commercial tetraploid cultivars with the aim of generating triploid or tetraploid progeny that exhibit the gigantic traits of polyploid plants^10^. Hundreds of cultivars, or hybrids, including amaryllis ‘Boca’, ‘Jax’, ‘Miami’, ‘Orlando’ and ‘Tampa’, have been bred widely for various types of pot plant production, cut flowers, rockery landscape plants, and greenhouse and garden use worldwide. Nevertheless, despite the benefits discussed above, this perennial flowering plant has yet to reach its full potential and has even declined in diversity in recent years. Although the development of hybrid breeding and cultivation has led to large cultivar variations, the combined effects of several biotic and abiotic factors^11,12^ as well as confusion over the taxonomy, phylogenetic relationships, and natural resources of this important plant mean that its genetic background still remains poorly understood. An advisable resource-based national development strategy has not been initiated to date. Thus, further information regarding the genetic diversity and population structure of amaryllis hybrids and cultivars is urgently required.

Genetic diversity and population structure analyses are primarily based on molecular markers, which are also very powerful tools for accelerating ornamental flower bleeding. In the case of amaryllis, random amplified polymorphic DNA (RAPD) and inter-simple sequence repeat (ISSR) are two examples of low-cost and simple markers that have been successfully applied to DNA fingerprint construction and genetic diversity assessment^13–15^. However, these approaches are not ideal for analyses of genetic diversity or population structure because they cannot easily be reproduced and are labour-intensive^16^. The use of simple sequence repeats (SSR) and microsatellite markers is preferable for research in this area as these sequences are abundant, multi-allelic, highly polymorphic and co-dominant^17^ and have been applied in genetic mapping, kinship studies, and cultivar identification^18^. Indeed, SSRs have proved to a valuable resource for probing polymorphisms in numerous plants. Additional genomic sequence data for amaryllis will also be useful for a broad range of genomic applications, as such co-dominant markers are important in genetic diversity analyses^19^. Mining reliable SSRs for agroforestry species is also vital for the assessment of genetic diversity and population structure to direct protection efforts and trace the filiation of accessions^20^. However, this process remains slow for most non-model species, and deficiencies in suitable SSRs have impeded genetic diversity and population structure analyses, including determining the history of domestication and the evolutionary relationships of *Hippeastrum*. A powerful tool for developing these markers is therefore critical if research on these themes is to progress.

Transcriptome sequencing, especially use of the Roche 454 and Illumina platforms, provides promising opportunities for high-throughput functional genomic research. Indeed, next-generation sequencing technologies have greatly reduced both the time and expense required to generated transcriptomic and genomic data. Read lengths obtained from both these platforms have recently been applied successfully to *de novo* transcriptome assembly in a number of economically important plants within Amaryllidaceae, including *Lycoris aurea*^21^*, L*. *longituba*^22^, and *L*. *sprengeri*^23^. These studies have clearly established that transcriptome sequencing provides a high-efficiency tool for developing large numbers of gene-based SSR markers. No favourable equivalent is available for generating genomic information in previously uncharacterized systems; this technique can also help discover the molecular mechanisms of functional genes and provide a simple approach for identifying molecular markers^24^.

We describe the generation, *de novo* assembly, and annotation of transcriptome datasets derived from buds of amaryllis cv. Blossom Peacock in this study. Approximately 27,273,776 high-quality clean reads were generated, and 171,462 *de novo* assembled unigenes were obtained. Once these unigenes were annotated, we developed 2,000 primer marker pairs that were screened to determine the genetic diversity and population structure of 104 amaryllis accessions. The results of this study include the first transcriptome sequence and SSR dataset for amaryllis and therefore provide a framework for future research on this plant.

## Results

### Illumina paired-end sequencing and de novo assembly of the amaryllis transcriptome

Sequencing the amaryllis cv. Blossom Peacock complementary DNA (cDNA) library using the Illumina HiSeq2000 platform generated a total of 28,974,793 raw reads comprising 5.85 Gbp of nucleotides. Subsequent to processing via strict filtration to trim adaptors and remove low-quality sequences, a total of 27,273,776 high-quality clean reads remained. The *de novo* assembly of 380,731 transcripts ultimately yielded 171,462 unigenes with a total length of 84,359,807 bp (Table 1) (mean = 492 bases, maximum = 8,272 bases, minimum = 201 bases) and an N50 of 610 bases using Trinity software^25^. The majority (74.69%; 128,058) of these unigenes had read lengths between 200 and 500 bp. In addition, 14.66% (25,138) of these unigenes had read lengths between 500 and 1,000 bp, and 10.65% (18,266) had read lengths greater than 1,000 bp.

**Table 1:** Summary of transcriptome statistics and functional annotations for amaryllis cv. Blossom Peacock.

Data type	Number
Total number of raw reads	28,974,793
Total number of clean reads	27,273,776 (94.13%)
Total number of transcript isoforms	380,731
Number of non-redundant unigenes	171,462
Number of unigenes between 200 bp and 500 bp in length	128,058 (74.69%)
Number of unigenes between 500 bp and 1,000 bp in length	25,138 (14.66%)
Number of unigenes greater than 1,000 bp in length	18,266 (10.65%)
N50 length (bp)	610
Total length (bp)	84,359,807
Maximum length (bp)	8,272
Minimum length (bp)	201
Average length (bp)	492
Number of unigenes in the NR database	47,359 (27.62%)
Number of unigenes in the Swiss-Prot database	27,089 (15.80%)
Number of unigenes in the COG database	14,151 (8.25%)
Number of unigenes in the GO database	35,153 (20.50%)
Number of unigenes in the KEGG database	18,048 (10.53%)
Number of unigenes annotated in at least one database	47,359 (27.62%)

### Sequence annotation

To further elucidate the potential functions of assembled unigenes, all 171,462 unigenes were subjected to blast searches against diversity databases (Table S1). Thus, transcriptome sequences were initially searched against the National Center for Biotechnology Information (NCBI) non-redundant nucleotide and Uniprot Swiss-Prot protein databases using BLASTX. The search was executed with a cut-off E-value of 10^−5^ and a significant similarity greater than 30%. The results of this comparison revealed that 47,359 (27.62%) and 27,089 (15.80%) of amaryllis cv. Blossom Peacock unigenes exhibit homology in the Nr and Swiss-Prot databases, respectively (Table 1).

**Figure 1:**
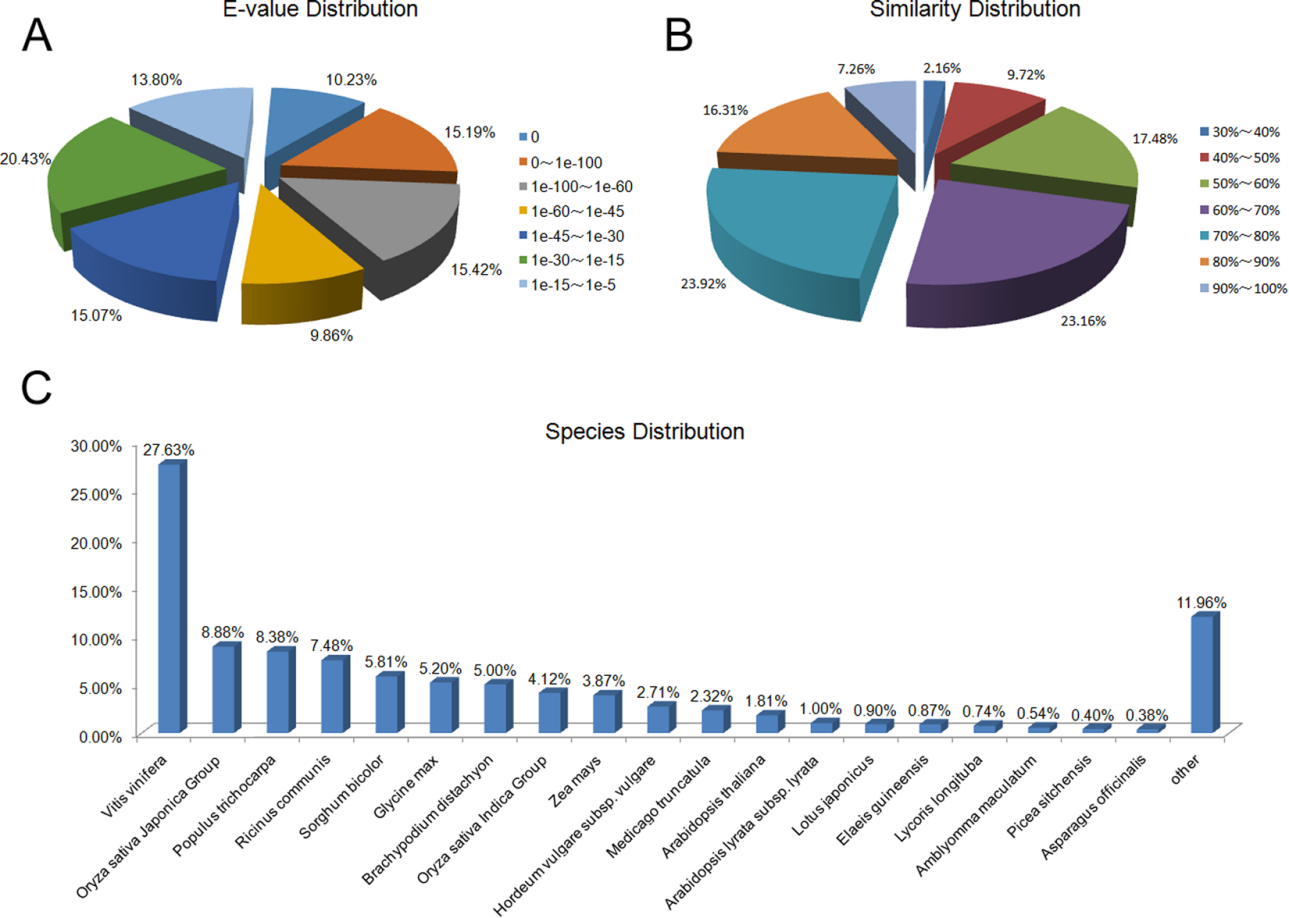
Features of amaryllis cv. Blossom Peacock unigene homology searches versus non-redundant protein databases (Nr) (E-value = 1E-10^−5^). (**A**) E-value distribution of BLASTx hits for each assembled unigene. (**B**) Similarity distribution of BLASTx hits for each assembled unigene. (**C**) Species-based distribution of the top BLASTx hits for each assembled unigene.

The E-value distribution revealed by these comparisons reveals that 15.19% of unigenes yielded significant hits (i.e., greater than 1E-100) in the NCBI non-redundant nucleotide database (Fig. 1A), whereas approximately 23.57% of unigenes exhibited greater than 80% similarity (Fig. 1B). The annotated transcriptome sequences of amaryllis share the highest degree of similarity with ‘*Vitis vinifera*’ (13,086; 27.62%) and the *‘Oryza sativa* Japonica Group’ (4,205; 8.88%), whereas the lowest level of shared similarity was noted for ‘*Populus trichocarpa*’ (3,967; 8.38%) (Fig. 1C).

**Figure 2:**
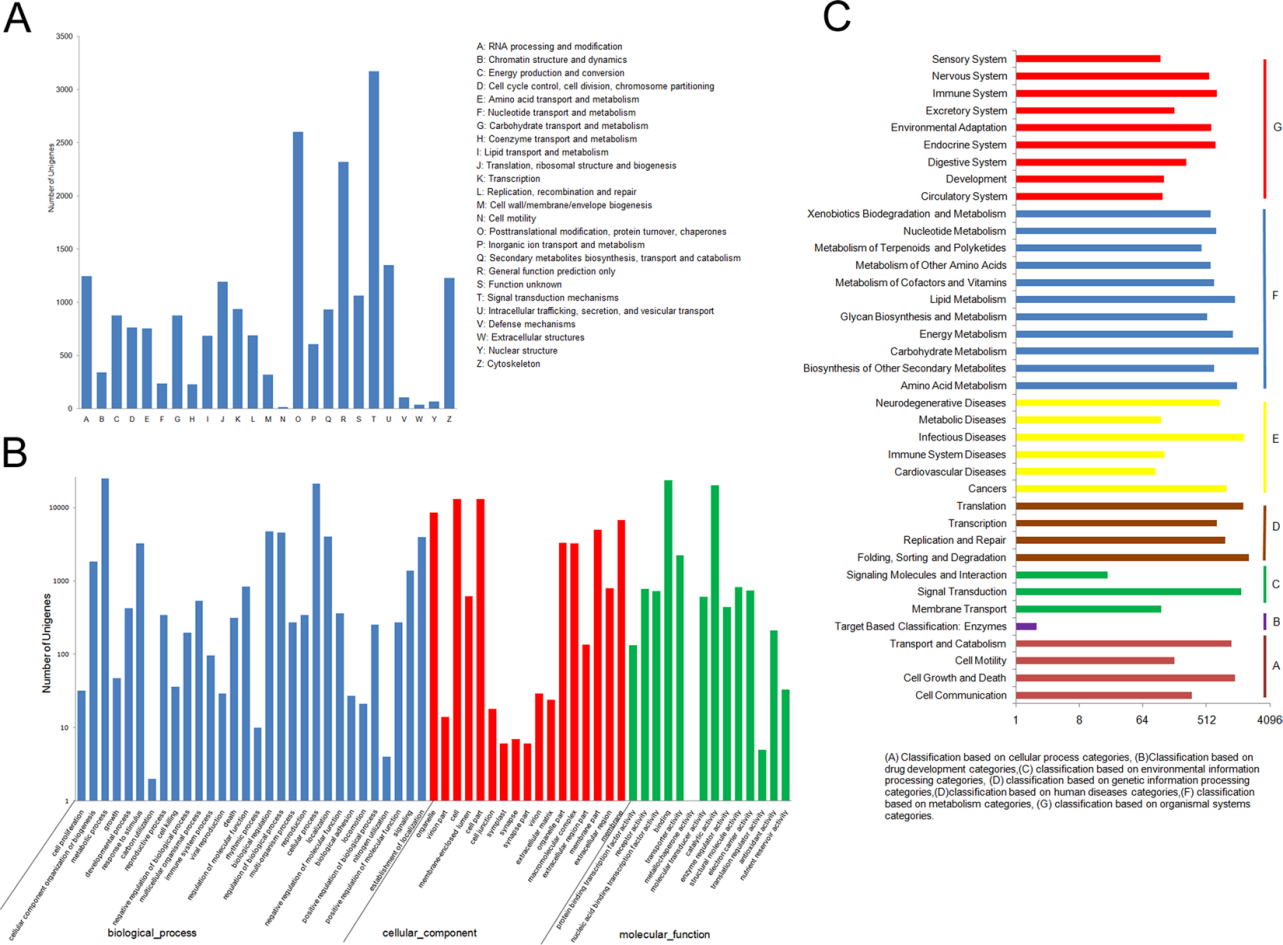
Classification of amaryllis cv. Blossom Peacock unigenes. (**A**) COG classification. A total of 14,151 assembled unigenes were annotated and assigned to 25 functional categories in this case. The *x*-axis denotes subgroups within the COG classification, whereas the *y*-axis indicates the number of genes in each main category. (**B**) GO classification of assembled unigenes at level 1. In this case, a total of 35,153 unigenes were grouped into three main GO categories: ‘biological processes’, ‘cellular components’, and ‘molecular function’. The *x*-axis denotes the subgroups within the GO annotation, whereas the *y*-axis indicates the percentage of specific gene categories. (**C**) The top 38 KEGG metabolic pathways for assembled unigenes. In this case, the x-axis denotes the number of genes in each metabolic pathway, whereas the y-axis indicates subgroups.

Additional blast searches revealed matches for 35,153, 14,151, and 18,048 unigenes in the Gene Ontology (GO), clusters of orthologous groups of proteins (COG), and Kyoto Encyclopedia of Genes and Genomes (KEGG) databases, respectively. Thus, to further evaluate the functions of amaryllis unigenes, 35,153 were matched with the GO database, and 74,604 were categorized based on these terms as the same unigenes are often mapped to different nodes. Comparisons reveal that these unigenes can be classified into three main GO groups: ‘biological processes’ (74,604), ‘cellular components’ (54,663), and ‘molecular functions’ (50,472). Within the first group, unigenes in the ‘metabolic processes’ category were most abundant (25,045; 71.25%), followed by ‘cellular processes’ (21,272; 60.51%). Of the 50,472 unigenes annotated in the ‘molecular functions’ category, 37.33% (13,122) were annotated within the ‘cell and cell parts’ category. Annotated unigenes in the ‘binding’ (23,614; 67.17%) and ‘catalytic activity’ (20,135; 57.28%) categories comprised the most common types of molecular functions (Fig. 2B; Table 1), whereas 14,151 of the total 171,462 non-redundant unigenes of amaryllis cv. Blossom Peacock unigenes were classified on the basis of 25 COG analysis. Within these vital sequences, the most abundant annotated group constituted ‘signal transduction mechanisms’ (3,172; 22.42%) followed by ‘post translational modification’, ‘protein turnover’, and ‘chaperones’ (2,605; 18.40%) as well as ‘general function prediction’ (2,321; 16.40%). A total of 1,062 (7.50%) were categorized as ‘function unknown’, 35 (0.25%) as ‘extracellular structures’, and 15 (0.11%) as ‘cell motility’ (Fig. 2A; Table 1).

To identify the biological pathways represented by the amaryllis unigenes assembled in this study, we compared our data with those in the KEGG database. A total of 18,048 unigenes could be assigned to 301 pathways from this database that consist of seven categories: ‘cellular processes’, ‘drug development’, ‘environmental information processing’, ‘genetic information processing’, ‘human diseases’, ‘metabolism’, and ‘organismal systems’. The KEGG group containing the largest number of amaryllis unigenes was ‘carbohydrate metabolism’ (748) within the ‘metabolism’ category followed by ‘fold, sort, and degradation’ (2,073) and ‘translation’ (1,705) within the ‘genetic information processing’ category as well as the ‘infectious diseases’ (1,737) group within the ‘human diseases’ category (Fig. 2C and Table 1).

### Search for flower developmental genes

We searched the presence of 269 candidate genes known to be involved in flower determination and development. In addition, we found that a portion of unigenes was assigned to the anthocyanin biosynthetic pathway (125), carotenoid biosynthesis pathway (30), specification of floral organ identity (13), photoperiod pathway (32), vernalization pathway (8), gibberellic acid pathway (9), ethylene biosynthesis pathway (22) and other genes of flower development (30). All of these unigenes are listed in Table S2. All of these unigenes may be involved in flower-related biological processes, such as flower development and formation. In amaryllis, the identification and analysis of these key genes will lay the foundation for understanding the potential molecular genetic mechanisms that control different aspects of amaryllis flower development in the future.

### The frequency distribution of SNPs

We assessed 66,4091 putative single nucleotide polymorphisms (SNPs) (Table 2 and Table S3). Among these SNPs, 423,307 were transitions (Ts), and 240,784 were transversions (Tv) with a Ts to Tv ratio of 1.76:1. The overall frequency of all SNPs was one SNP per 7.87 kb. The most abundant SNPs detected were C/T (212,902, 32.0%) followed by A/G (210,405, 31.7%), A/T (76,217, 11.5%), A/C (60,232, 9.1%), T/G (57,440, 8.6%) and C/G (46,895,7.1%). The frequencies of A/C, T/G and C/G were close, with each being less than 10%.

**Table 2:** Summary of SNPs identified from unigenes of amaryllis cv. Blossom Peacock.

Transitions	Number	Transversions	Number
C/T	212,902	A/T	76,217
A/G	210,405	A/C	60,232
		T/G	57,440
		C/G	46,895
Total	423,307	Total	240,784

### The frequency distribution of SSRs

**Figure 3:**
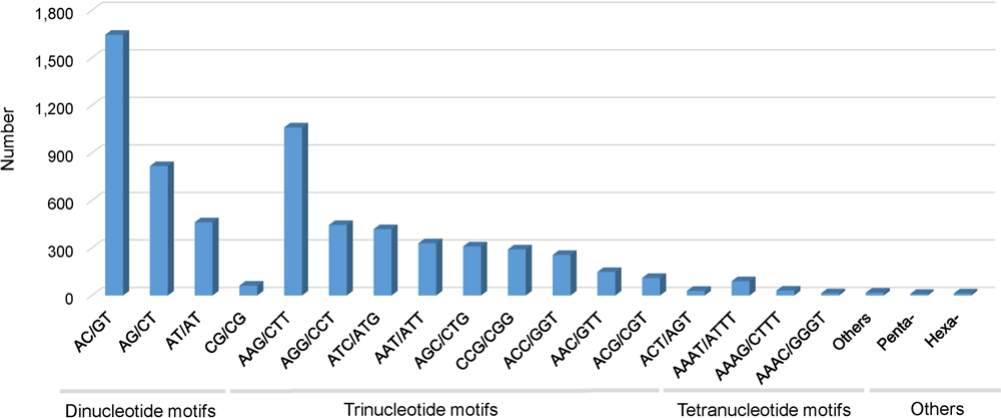
Summary of SSRs identified in amaryllis cv. Blossom Peacock unigenes.

A total of 171,462 unigenes were employed for further analysis using the default parameters in the software MISA, including a total of 6,599 di-, tri-, tetra-, penta-, and hexanucleotide SSR motifs (i.e., five, four, four, four, and four repeat numbers, respectively). Trinucleotide motifs comprised the most abundant type of repeats (3,400; 51.52%) within our sample of amaryllis unigenes followed by di- (2,987; 45.26%), tetra- (189; 2.86%), hexa- (13; 0.2%), and pentanucleotide motifs (10; 0.16%; Fig. 3). The most abundant repeats revealed by this analysis were AC/GT (1,646; 25.08%) and AAG/CTT (1,061; 16.17%) followed by AG/CT (817; 12.45%), AT/AT (462; 7.04%), AGG/CCT (445; 6.78%), ATC/ATG (419; 6.38%), and AAT/ATT (330; 5.03%; Fig. 3). Similarly, the most abundant tetra-repeat motif types recovered by this analysis were AAAT/ATTT (90; 1.37%), whereas the total number of hexa- and pentanucleotide motifs was 10 (0.16%) and 13 (0.2%), respectively. The distribution of SSRs within the amaryllis cv. Blossom Peacock genome can therefore be estimated to be one every 4.2 kb.

### Genetic diversity of SSR loci

Among a total of 6,599 SSR loci, 6,460 loci with appropriate sequences were reserved for marker design, and a total of 335 SSR loci were randomly selected for the synthesis of PCR primer pairs containing 129 di-, 142 tri-, 64 tetranucleotide repeats. In an initial screen of 335 SSR primers (Table S4) from 17 accessions (i.e., Red Lion, Ferrari, Carina, Rilona, Fairytale, Desire, Bolero, Blossom Peacock, Exotic Peacock, Dancing Queen, Double Dragon, Alfresco, Flame Peacock, Aphrodite, Intokasi, and Red Peacock), 154 (43.6%) primer pairs successfully amplified polymorphic bands, whereas the remaining 199 (56.4%) failed to generate either amplification or polymorphic products. Thus, from the 154 primers that amplified, 21 polymorphic, easily scorable SSRs were selected to assess the genetic diversity and relationships of 104 *Hippeastrum* accessions (Table 3). Analysis of these SSRs enabled the identification of 98 alleles across all microsatellite loci. The mean number of alleles per locus was 4.67, ranging between 3 (i.e., FP033, FP083, FP131, FP220, and FP255) to 8 (i.e., FP089 and FP257). The mean number of polymorphic loci was 4.38, ranging between 1 (FP105) and 8 (FP089, FP257). The mean Nei’s gene diversity (H) of these markers was 0.264, ranging between 0.055 and 0.394. The average Shannon’s Information index (I) value was 0.41, ranging between 0.172 and 0.567. FP083 and FP249 markers exhibited the lowest and highest genetic diversity, respectively (Table 3).

**Table 3:** Genetic diversity estimates based on 21 SSR primers from 104 amaryllis accessions.

Locus	Motif	Primer sequences (5′-3′)	Length (bp)	N	NP	H	I
FP027	(TGT)7	F:CCAAATGATCCCAAGGAAGA	184–196	4	4	0.380	0.561
		R:TCATGACACCTTCGGAGACA					
FP033	(GAG)7	F:GAGTCGAGGTGGGTGTTGAT	127–140	3	3	0.380	0.567
		R:ATCTCCCCAAACCCCATAAG					
FP047	(AGA)7	F:TGAATGTGTTTGAGGCTTGC	164–220	7	6	0.163	0.269
		R:TTGATCCTCTTTTCTTGTGGC					
FP077	(CCCT)5	F:GGCATTATCACGCCTAAGGA	146–212	4	3	0.265	0.401
		R:CACCACAAGAAACCGAACAA					
FP083	(TTTA)5	F:CCAACTGTAAGAAACCCCCA	183–195	3	3	0.055	0.121
		R:CCCAAAGGCCTAAATTCACA					
FP089	(GGAG)5	F:GAGGATGCACTCTTTGAGCC	192–300	8	8	0.293	0.438
		R:CGTCAACTCCTCTTCCTTCG					
FP105	(GAA)7	F:CGGTGGGAGAAGAAGAGATG	242–269	4	1	0.124	0.172
		R:GAGACGATGAAGCTCCGAAT					
FP115	(TA)10	F:CGGGTCAATGTTAAGCCAGT	147–181	5	5	0.193	0.323
		R:CAGGTGATGAGCATTGGATG					
FP116	(AG)7-(AG)7	F:TCGGGGCAGACATCTTTAAC	145–167	4	4	0.195	0.332
		R:GCTTTGGGAGGTATTTTTGTGA					
FP131	(AG)10	F:TCGAGGTGCTGTTTGTTTTG	125–133	3	3	0.316	0.481
		R:AGACCAACGCAAGTCAGTCC					
FP136	(GAG)8	F:GAGCTTGACCTGACGGACTC	175–190	5	5	0.271	0.416
		R:GCAGAGCATGGCTTCTATCC					
FP213	(TTTA)5	F:CCCCTTTTGTAGATGCCTCA	116–132	5	5	0.256	0.405
		R:AATTGAGACAGGCGTTTTGC					
FP215	(ACC)7	F:TCGCTTCTCCAATCTCGACT	98–113	6	6	0.258	0.410
		R:GTCGATCGCAACCATTCTTT					
FP220	(GC)6-(TG)6	F:TGCCATTTAAGATCAATGGAAG	140–148	3	3	0.287	0.449
		R:AAGTGGGCAGCTGAAAAAGA					
FP249	(CTT)7	F:TGTGGGTTCTATGCTTTCCC	131–146	5	5	0.394	0.568
		R:CCCCTGCTTCATCTCCAATA					
FP255	(ATTT)5	F:AGGAAATCATTGGAGACCGA	145–154	3	3	0.344	0.499
		R:AATATAGCCCCTCTCACCCC					
FP257	(CT)10	F:CAGCGCTCTTGCTCAGTAGA	137–167	8	8	0.214	0.354
		R:ACTCAGGGTCATGAAAACGG					
FP259	(CT)8-(CA)7	F:TCTCCAAAACCTTCTTCTCACA	116–126	4	4	0.392	0.577
		R:CTCGAGGAGGAGAGATGGGT					
FP280	(AATA)5	F:TGAACAGTGAAACTCGGCAG	166–194	6	6	0.379	0.558
		R:TGTGGTGGAAATTTTCTTCATT					
FP292	(AATA)5	F:GATGCAAGAAGGGTTCCAAA	110–122	4	3	0.126	0.229
		R:TTGCATTTTAACAGCGCAAG					
FP305	(CAA)7	F:CACCATGCCAACCTTCTTCT	165–175	4	4	0.265	0.421
		R:CCTGCTGAGATTTTGCCTTC					
Total				98	92	5.550	8.550

Note: N: Number of loci; NP: Number of polymorphic loci; H: Nei’s gene diversity; I: Shannon’s Information index.

### The genetic relationships and population structure of cultivated amaryllis

**Figure 4:**
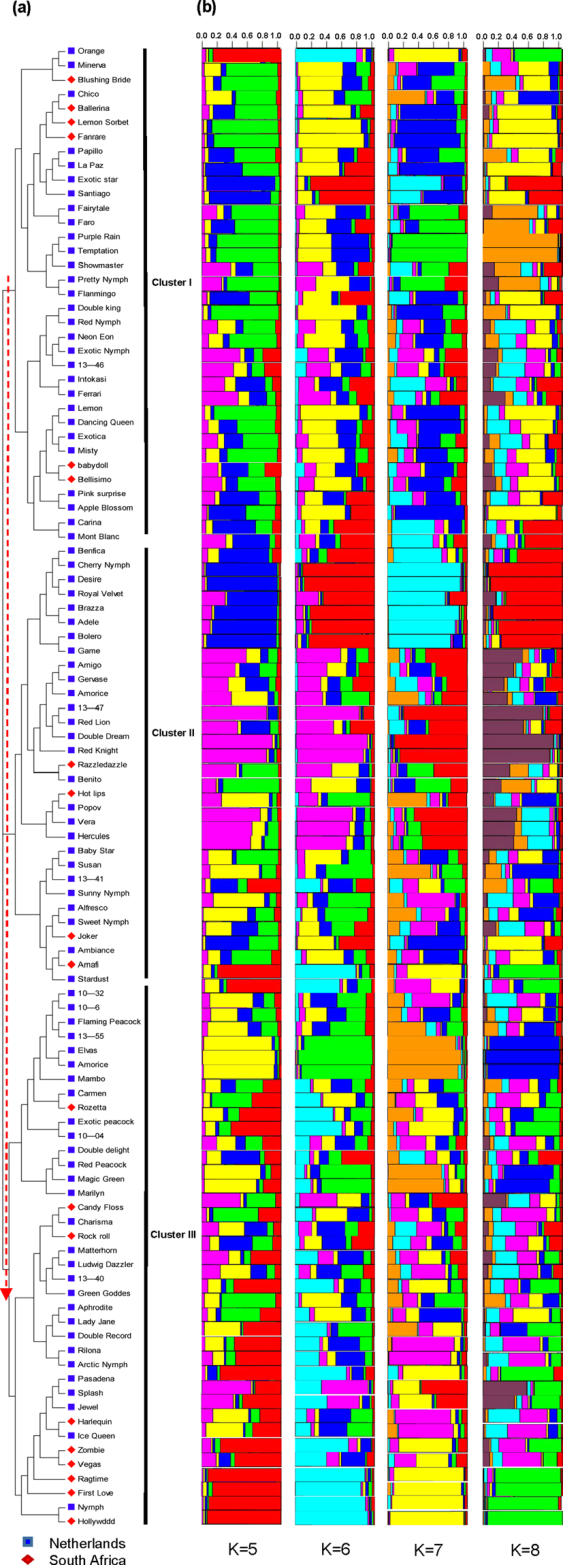
N-J phylogenetic tree aligned with a structural analysis of 104 amaryllis accessions from the Netherlands and South Africa. (**a**) N-J tree for 21 SSR markers based on H values depicting three major clusters, including I (35 accessions), II (31 accessions) and III (38 accessions). Each of these clusters was further separated into two sub-clusters. (**b**) Multilocus cluster analysis using STRUCTURE software, demonstrating the four best fitting models (i.e., *K* = 5, *K* = 6, *K* = 7, and *K* = 8) on the basis of Evanno’s D*K*. Each individual accession on this figure is represented by a vertical line divided into *K* coloured bars.

We constructed a neighbour-joining (N-J) tree using the software MEGA to hypothesize the genetic relationships amongst 104 *Hippeastrum* accessions. The resultant dendrogram demonstrates that all amaryllis cultivars and hybrids can be obviously separated from one another. These cultivars and hybrids are denoted cluster I (35 accessions), cluster II (31 accessions), and cluster III (38 accessions) (Fig. 4a). Each of these clusters is further separated on the N-J tree into two sub-clusters. For example, sub-cluster I within cluster I mainly comprises the 18 accessions between ‘Orange’ and ‘Flamingo’, 14 of which are from the Netherlands and four are from South Africa. Sub-cluster II primarily comprises the 17 *Hippeastrum* accessions between ‘Double king’ and ‘Mont Blanc’, which is composed of 15 accessions from the Netherlands and two from South Africa. Similarly, sub-cluster I within cluster II includes 21 accessions, 19 of which are from the Netherlands and two are from South Africa. Sub-cluster I is obviously distinct from sub-cluster II, which contains ten accessions, including eight from the Netherlands and two from South Africa. Finally, cluster III contains 38 accessions, 29 of which are from the Netherlands and nine are from South Africa. It is also noteworthy that the accessions within each cluster can also be described on the basis of their petal number (single or double) and colour, including ‘Chico’ and ‘Ballerina’ (both with double pink or red flowers) along with ‘Papillo’, ‘La Paz’, ‘Exotic star’, ‘Santiago’, ‘Fairy tale’, and ‘Faro’ (single petals) in cluster I. These accessions are distinct from ‘Benfica’ and ‘Cherry Nymph’ (double petals); ‘Bolero’ and ‘Faro’ (single and pink or red flowers); ‘Brazza’ and ‘Adele’ (single and red flowers); and ‘Amigo’, ‘Gervase’, ‘Amorize’, ‘13–47’, ‘Red Lion’, ‘Double Dream’ (single or double and pink or red) in cluster II. All of these varieties are distinct from ‘10–32’ and ‘10–6’ (double, red and white complex colour), which group with ‘Pasadena’, ‘Splash’, ‘Jewel’, ‘Harlequin’, ‘Ice Queen’, ‘Zombie’, ‘Vegas’, ‘Ragtime’, and ‘First Love’ (double peals) in cluster III.

The genetic structure of the 21 SSR markers determined for these 104 accessions was examined using the statistical model in the software STRUCTURE. This analysis yielded K values that range between 1 and 15, and the most likely number of groups was determined by calculating Delta K (ΔK). Thus, the highest ΔK values were observed when K = 5 (i.e., ΔK = 51.92), K = 6 (i.e., ΔK = 41.17), K = 7 (i.e., ΔK = 44.54), and K = 8 (i.e., ΔK = 33.35). These results suggest that all accessions can be separately classified into five, six, seven, and eight sub-groups, respectively (Fig. 4). The genetic structure of each accession is illustrated using a bar plot to enable a simple comparison with the N-J tree (Fig. 4).

## Discussion

Although amaryllis represent an economically important plant group that has become increasingly influential in research on ornamental flowers, a general lack of information about the SSR markers within their transcriptome has hampered genetic research. The results presented here are consistent with previous studies that have developed limited genetic resources for other members of Amaryllidaceae^21–23^ and demonstrate that transcriptome analysis and marker development is possible for this flowering plant.

### Developing genetic resources for amaryllis through transcriptomics

Transcriptome sequencing studies are highly valuable for the discovery of molecular markers as well as characterization and variant analysis as components of the identification of novel genes^24^ and provide the basis of our knowledge of genome intricacy. Transcriptome diversity is significant in the case of flowering plants, such as amaryllis, that have very large genomes and therefore represent a sequencing challenge. A total of 27,273,776 clean reads obtained from the Illumina HiSeq2000 platform were used to assemble the transcriptome of *H*. *hybridum* cv. Blossom Peacock in this study. Of note, the Roche 454 platform was initially favoured for transcriptome assessment in this research given its larger read length, the possibility to equalize for deficiencies in the reference genome, and the fact that it was used successfully for *L*. *aurea* sequencing in a previous study^21^. Although Illumina platform sequencing is particularly limited in some aspects, recent procedural developments in assembly have enabled high-quality *de novo* assembly for a number of species, including *L*. *sprengeri*^23^ (Amaryllidaceae). This study further illustrates successful *de novo* assembly in amaryllis combined with analysis of diversity data and standards on the basis of 380,731 transcripts representing 171,462 unigenes from 28,974,793 raw reads (Table 1). This result is significant because the average length of unigenes is generally assumed to be a sign of high-quality assembly^26^. One obvious feature of our *H*. *hybridum* cv. Blossom Peacock assembly is the unigene average (610 bp), which is greater than the average length previously recovered for *L*. *sprengeri*^23^ (385 bp) using the Illumina HiSeq2000 platform. Comparisons with Roche 454 platform sequencing of *L*. *aurea* (329 bp)^21^ further confirms the effectiveness of our *H*. *hybridum* cv. Blossom Peacock transcriptome assembly. One possible explanation for the longer mean length recovered in amaryllis might be the broader sequencing coverage provided by the Illumina platform. In addition, sequence comparisons, annotation, and marker validation may also be important factors that contribute to the quality of *de novo* assembly.

Results indicate that the amaryllis sequences assembled in this study are diverse compared with those from existing similar data and that 47,359 (27.62%) and 27,089 (15.80%) unigenes exhibit significant homology versus the Nr and Swiss-Prot databases, respectively. These observations suggest that sequence contiguity remains consistent over parts of the annotated transcriptome; thus, unigenes that do not match with existing databases might either be indicative of novel amaryllis-specific genes or correspond with non-coding regions, pseudogenes, or short transcript lengths. Similar BLAST-based methods have been utilized in previous studies. For example, 45,052 (45.9%) *L*. *sprengeri* unigenes and 66,197 (46.91%) *L*. *aurea* unigenes were matched significantly with known counterparts in public databases. This finding suggests that the amaryllis sequence overlaps considerably with previously collected data.

Several methods were utilized in this study to analyse the biological function of assembled transcripts, including GO, COG, and KEGG. The GO analysis results revealed that 20.50% of unigenes proceeded through this annotation and that it was a beneficial approach for determining the diversity of gene functions. To gain further insights on specific metabolic and cellular processes controlled by gene functions in the context of genetically and biologically complex behaviours^27^, we mapped 8.25% of our annotated unigenes against the COG database and 10.53% to KEGG terms. These results are similar to those previously reported for *L*. *sprengeri* and *L*. *aurea* and indicate that the transcripts generated in this study for *H*. *hybridum* cv. Blossom Peacock are high quality and might be applicable to future studies that address amaryllis genetic cloning, molecular heredity, and transgenesis.

In addition, single-gene annotation and predictive pathways have accelerated the discovery of key genes associated with flower development and function of amaryllis flowers. All in all, we obtained 269 genes related with eight pathways, including anthocyanin biosynthetic pathway (125), carotenoid biosynthesis pathway (30), specification of floral organ identity (13), photoperiod pathway (32), vernalization pathway (8), gibberellic acid pathway (9), ethylene biosynthesis pathway (22) and other genes of flower development (30). These special unigenes indicated that relatively accurate and high mean genome databases can be generated through de novo transcriptome analysis of non-model plant species.

### Detection and validation of amaryllis SNP and SSR markers

The use of SSRs and microsatellite markers have a number of advantages compared with other systems and are thus of considerable importance to our understanding of genetic diversity, map construction, comparative genomics, and molecular breeding^28^. Identification of a number of SSRs was therefore undertaken as part of this study to enrich the known genomic resources of amaryllis. The mean frequency detected in *H*. *hybridum* cv. Blossom Peacock corresponds with one SSR locus per 4.2 kb, which is greater than that previously reported for coloured calla lily (*Zantedeschia* hybrid)^29^ (i.e., one SSR locus per 7.27 kb) but lower than that for *Zantedeschia rehmannii*^30^ (i.e., one SSR locus per 4.1 kb). These differences may be due to the use of different standard repeat units and length thresholds^31^, the number of data libraries searched, and SSR identification instruments^32^. We also determined that 51.8% of our total SSRs are trinucleotide repeats followed by dinucleotide (45.5%), and tetranucleotide repeats (2.3%) as well as pentanucleotide and hexanucleotide repeats, which collectively comprise 0.4% of the motifs. One important characteristic of amaryllis appears to be that the frequency of motif repeats simultaneously decreases in contrast to that of *Z*. *rehmannii*^30^. Thus, compared with some other plants^33^, our results suggest that trinucleotide repeats are the most common genetic motif of this type in amaryllis. Within these repeats, AAG/CTT is most abundant (31.2%). Further, among a total of 335 SSRs, 154 primer pairs successfully amplified polymorphic bands in *Hippeastrum* accessions. The proportion is not very high. This low amplification and polymorphism rate in 17 accessions may be due to the presence of introns in primers, assembly errors, and the high heterozygosity of the amaryllis genome. We selected a total of 21 primer pairs, almost all of which were able to provide single, clear amplification products in all 104 varieties. This result suggests that the SSRs sequenced in this study are sufficient to determine the relationships amongst the accessions used in this study despite the fact that the short and robust nature of these repeats in amaryllis has previously prevented a clear evaluation of genetic diversity.

Single nucleotide polymorphisms (SNP) have recently become a more popular marker for high-density genetic mapping, association mapping and population genetic structure studies^34^. To understand the SNPs of *H*. *hybridum* cv. Blossom Peacock, we detected SNPs of *H*. *hybridum* cv. Blossom Peacock. The mean frequency detected in this cultivar corresponds with one SNP locus per 7.87 kb, which is greater than one SSR locus in this report. On the other hand, this finding may indicate the polyploidy or genetic complexity of the Hippeastrum variety.

We resolved this issue in this study by mining novel microsatellite loci that will expedite a more comprehensive assessment of amaryllis in future research.

### Assessing the genetic diversity of amaryllis accessions

As molecular markers, SSRs are of great importance to the assessment of genetic diversity. In total, 21 primer pairs were selected for further PCR validation in this study. Our results clearly elucidate the genetic diversity among amaryllis cultivars and hybrids and reveal the effectiveness of our mined markers because significant H and I values were obtained and amaryllis accessions could be clearly distinguished. The number of polymorphic loci (94%) across all 21 primers detected in this study is increased compared with previous studies focused on the RAPD^13^ (72.6%) and ISSR^15^ (92.4%) of amaryllis accessions. However, both H and I values might be useful to distinguish accessions to a certain extent (Table 3). Average H and I values in these cases were 0.26 and 0.41, respectively. These results however may not have reached the desired level for significant comparisons with previous ISSR^15^ and RAPD-based^13,14^ diversity studies in amaryllis in part given the higher number of samples. In addition, SSR loci also occurred at low frequencies in some markers, including FP083 (0.055, 0.121) and FP105 (0.124, 0.172), which is consistent with the results of previous studies^35^. One reason for a lower number of alleles is possibly the fact that these accessions belong to different species, which might lead to an increased number revealed by a specific SSR marker^36,37^. In addition, species breeding behaviour, collection genetic diversity and size, the sensitivity of the genotyping method and the genomic locations of markers could all influence the data included in this research.

### Cluster analysis and genetic structure within *Hippeastrum* spp. accessions

Some previous studies have regarded the number of amaryllis flowers (i.e., single or double) and colour as key reference standards to perform clustering and structural analysis given that a significant amount of breeding is performed based on these traits^38,39^. McCann presented the first discussion of an amaryllis double flower in 1937 on the basis of a wild type *Amaryllis puniceum* specimen collected from Cuba. Six known petal sources at the possible origin of accumulations, bracts, male and female stamen, taiwan pavilion, repetition, and inflorescence are known for most plants^40^. In a previous study, hereditary observations revealed that an increase in the number of amaryllis petals is derived from stamens in male and females and that the basal trait is dominant^40^. Given that previous studies have also demonstrated that the ABC and four factor models both determine the development of floral organs^41^ within the same growth environment, both flower number and colour are most likely genetically regulated and can therefore be reliably reconstructed using clustering based on these traits.

However, the N-J tree and structural plot generated in this study (Fig. 4) demonstrate that the 104 accessions we considered cannot be fully clustered according to the number of flowers or their colour. Exceptions include the ‘Lemon sorbet’, ‘Ballerina’ and ‘Chico’ varieties. Of these varieties, the former possesses green single petals, whereas ‘Ballerina’ (red flowers) and ‘Chico’ (pink flowers) both have double petals. These exceptions are important because they illustrate the comprehensive nature of amaryllis population structure.

We believe that there are two main explanations for the complex population structure of amaryllis. The first explanation involves the abundance, wide distributional range, and long history of species breeding within this plant group. Amaryllis are native to the mountainous areas of South America, including Brazil, Peru, Argentina, and Bolivia^42^, and this ornamental plant has been bred in the UK since the 1690s. Although the first recorded documentation of *Hippeastrum* hybrids was in 1799 in the UK as ‘Johnsonii’ (*H*. *vittatum* X *H*. *regime*), the introduction of these central and South American flower crops into Europe occurred later during the 19^th^ century. Thus, after numerous breeding experiments, *Hippeastrum* was divided into four key hybrid groups: Reginae, Leopoldii, Vittatum and Reticulatum^9^. Amaryllis gradually became a very popular flower in the West with greater than 300 cultivars^5^. As the cultivation of amaryllis and its hybrids is now observed in many countries around the world, including the Netherlands, South Africa, Japan, Brazil, and the USA, the original founder species has lost genetic relationships amongst many cultivars and hybrids due to long-term artificial cultivation and breeding. An additional reason for this confusion is the existence of a polyploid variety of amaryllis, *H*. *hybridum*, a bulbous perennial with ploidy level that ranges between diploid and octoploid^2^. Examples include *H*. *aulicum*, *H*. *machupichense*, *H*. *psittacinum*, and *H*. *solandrifoliu* (2n = 22); *H*. *forgeti* (2n = 2x + 1 = 23) and *H*. *argentinum* (2n = 33); *H*. *reginae* (2n = 44); and *H*. *rutilum* (n = 55)^43^. As a direct result of this diversity, amaryllis has gradually become an increasingly popular flower globally. This polyploidy has resulted in a large floral organ and a high yield, which have both attracted researchers. Thus, a big flower has always been a clear goal of breeding experiments^38^. These characteristics also indicate that the complex structure of amaryllis relationships have proved difficult to untangle. The tree-based clustering and structural analysis presented in this study do not reveal any obvious associations with the geographic origin of accessions, but this finding might also be related to the fact that accessions have only been derived from the Netherlands and South Africa. Further studies involving larger samples derived from extended geographical regions are required to develop more generalized conclusions regarding the divergence and population structure of *Hippeastrum* accessions.

The aim of this study was to develop and assign SSRs to elucidate the genetic diversity of 104 *Hippeastrum* accessions. We confirmed the potential of transcriptome sequencing in the case of species where genomic information is lacking such as *Hippeastrum*. SSR resources have also been shown to be useful for genetic detection and localization of this species and may help improve the plant’s floral quality.

## Materials and Methods

### Plant material and DNA extraction

The 104 amaryllis cultivar and hybrid specimens used in this study (Table S5) (85 from the Netherlands and 19 from South Africa) were housed in an experimental greenhouse at the Beijing Academy of Agriculture and Forestry Sciences, Beijing, China. All specimens were planted in fertile land containing a mixture of sand and soil (1:1, v/v) and were subjected to a completely randomized range of temperatures between 18 and 22 °C from April to November 2014. All accessions were grown under similar conditions and were irrigated and fertilized monthly. Fresh young leaves were collected in the spring, immediately frozen in liquid nitrogen and stored at −80 °C until they were used for DNA extraction. Total DNA was separately extracted from each specimen using a DNeasy Plant Mini kit according to the manufacturer’s instructions (Zexing Biotech, Beijing, China). The quality and quantity of genomic DNA was assessed via resolution in a 1% (w/v) agarose gel using a Qubit® 2.0 Fluorometer.

### RNA isolation, library preparation, Illumina sequencing and *de novo* transcriptome assembly

Sample flowers were also frozen directly in liquid nitrogen and stored at −80 °C for RNA extraction. Total RNA from each sample was extracted using an RNeasy Plant Mini Kit (Zexing Biotech, Beijing, China) according to the manufacturer’s instructions. The quality and quantity of RNA was assessed using a NanoDropND 2000C spectrophotometer (Thermo Scientific, USA) and Bioanalyzer (Agilent Technologies, USA). Poly (A)+ mRNA was enriched with magnetic beads cohering Oligo (DT)_25_, and equal quantities of poly (A)+ mRNA from each sample were mixed and used as modes to construct cDNA libraries. These libraries were synthesized using the standard Illumina pipeline (Illumina, San Diego, CA, USA) according to the manufacturer’s instructions at the Yuanquan Yike Biotechnology Company Limited (Beijing, China). The quality of raw reads was assessed using the software Trinity^25^ before *de novo* assembly was performed using sequence adapters following the removal of low quality reads for subsequent analyses. All Illumina sequencing reads for *H*. *hybridum* cv. Blossom Peacock were submitted to the NCBI Sequence Read Archive under the accession number SAMN05149972.

### Functional annotation and classification of unigenes

Functional annotation and assembled unigene classification followed the method previously described by Meyer *et al*.^44^. Assembled unigenes were searched to assess their similarity versus the NCBI Nr and Swiss-Prot protein databases using BLASTX^44^ with the threshold E value set to greater than e-10^–5^. BLAST results were then imported into Blast2GO (Version 2.5.0), and each sequence was assessed versus the GO database using default parameter settings. All unigene sequences were also aligned to the COG database to predict and classify their possible functions. Biological pathway information indicative of molecular interactions and reaction networks was annotated based on the KEGG pathway.

### Search for genes of interest

We searched all genes that were involved in anthocyanin biosynthesis pathway, carotenoid biosynthesis pathway, specification of floral organ identity, photoperiod pathway, vernalization pathway, gibberellic acid pathway, ethylene biosynthesis pathway and other genes of flower development (Table S2) from different species and used them to trace our annotated contigs by similarity to identify candidate genes that correlate with flower organ formation and double flower formation. These final results were used for further bioinformatics analysis^45^.

### The frequency distribution of SNPs

All unigenes were retained to identify potential SNPs using the MISA tool (MIcroSAtellite)^46^. SNPs were detection using the CLC Genomics Workbench 6.

### SSR marker verification in amaryllis accessions

All unigenes were retained to identify potential SSRs using the MISA tool (MIcroSAtellite), and a series of searches were performed to identify di-, tri-, tetra-, penta-, and hexanucleotide motifs with a minimum number of six, five, four, four, and four^47^, respectively. The software Primer Premier 5.0 (PREMIER Biosoft International, Palo Alto, CA) was then used to manually design 335 PCR primer pairs to select SSRs with greater than 20 motif loci, which were then synthesized at Sangon Biotech (Shanghai, China) and used to validate polymorphisms in 17 amaryllis accessions. A total of 154 SSR primers yielded significant and reproducible products from this step before 21 SSR primer pairs were randomly selected to assess the diversity of 104 amaryllis accessions (Table S5).

We performed PCR with each primer on DNA from all 104 accessions to amplify microsatellite-containing regions. Thus, PCR reactions (12.5 μl volume) were conducted using a GeneAmp PCR System 9700 (Applied Biosystems) with each reaction containing 4 μl of genomic DNA (5 ng/μl), 0.4 μl of each primer, 0.25 μl of high purity dNTPs, 0.2 μl of Taq10 DNA polymerase, 1.25 μl of 10 × Taq10 buffer I or II, and 6 μl of ddH_2_O. PCRs were conducted using the touchdown cycling program. Thus, an initial denaturation at 94 °C for five minutes was followed by 11 touchdown cycles between 60 °C and 50 °C, 45 seconds at 94 °C, annealing for 40 seconds at 60 °C (the annealing temperature for each cycle was reduced by 1 °C per cycle), and extension for one minute at 72 °C. This program was accompanied by 15 denaturation cycles at 94 °C for 45 seconds followed by annealing at 50 °C for 40 seconds, elongation at 72 °C for one minute, and ten minutes of extension at 72 °C before a final infinite hold at 4 °C. We performed electrophoresis on an 8% non-denaturing polyacrylamide gel (PAGE) with a Tris/borate/EDTA (TBE) buffer running at 150 V for one hour to separate PCR products before gels were silver stained as described in previous work on the coloured calla lily^29,30^. SSR markers amplified at sizes between 100 bp and 400 bp were converted into ‘0’ and ‘1’ codes denoting ‘absence’ and ‘presence’, respectively.

### Data analysis of genetic diversity and population structure

Values for N, NP, H, and I were calculated for each SSR locus using the software POPGENE 3.2^48^, and a N-J dendrogram comprising the 104 amaryllis accessions was generated using the software packages PowerMarker version 3.25 and MEGA 5^29^. The assignment of 104 SSR accessions to different clusters was then performed based on 21 genetic SSRs using the model-based software STRUCTURE v2.3.3^49^. This application analyses SSR marker data to determine the attribution of clusters. Thus, for each K value (ranging between 1 and 15), at least ten independent admixture ancestry model runs were accomplished employing a 200,000-iteration burn-in period followed by 200,000 subsequent iterations. The best value for K clusters was then calculated following the method of Gilbert *et al*.^50^, and the mean likelihood value, L (K), across all runs was estimated for each K-value. We used Delta K as our model choice criterion to indicate a clear peak for the most likely K-value, which was calculated using the formula Delta K = m(|L(K)|)/s[L(K)]^51^. We regarded the level of the Delta K value to be tantamount to a test of our structural analysis.

## Electronic supplementary material

Table S1

Table S2

Table S3

LaTeX Supplementary File

## References

[1] Bailey, L. H. Staff of Liberty Hyde Bailey Hortorium Hortus third (1976).

[2] Guerra M (1990). Cytogenetics of angiosperms collected in Pernambuco-III. Sci and Cul..

[3] RockweIl, F. F., Grayson, E. C. The Complete Book of Bulbs. *New York*. (1977).

[4] Sebben C (2015). New Lycosinine Derivative from *Hippeastrum Breviflorum*. Rev Brasi Farmacogn..

[5] Jamil MK, Rahman MM, Hossain MM, Hossain MT, Karim AS (2016). Influence of sucrose and aluminium sulphate vase life of cut Hippeastrum flower (*Hippeastrum hybridum* Hort.) as influenced. Bangladesh J Agri Res..

[6] Guo Y (2016). New alkaloids from *Hippeastrum papilio* (Ravenna) van Scheepen. Helv Chim Acta..

[7] Sultana J (2010). *In vitro* bulb production in Hippeastrum (*Hippeastrum hybridum*). J Cent Eur Agr..

[8] Vazquez C, Reed ST, Dunn C (2015). Nitrogen Fertilization as Ammonium or Nitrate-N on *Hippeastrum hybridu*m BulbGrowth. Agr Sci..

[9] Callaway, D. J., Callaway, M. B. Breeding ornamental plants. 174–195 (Timber Press, 2000).

[10] Meerow AW (2014). The Florida Series of Hybrid Amaryllis: Five New *Hippeastrum* Cultivars. Hortscience..

[11] Xu XH (2017). First Report of *Hippeastrum mosaic virus* in *Hippeastrum* spp. in Mainland China. Plant Dis..

[12] García N (2017). Deep reticulation and incomplete lineage sorting obscure the diploid phylogeny of rain-lilies and allies (*Amaryllidaceae* tribe *Hippeastreae*). Mol Phylogenet Evol..

[13] Phuong PTM, Isshiki S, Miyajima I (2014). Genetic variation of *Hippeastrum* accessions in Vietnam. J Fac Agr Kyushu U..

[14] Chakrabarty D, Gupta VN, Datta SK (2007). Varietal identification and assessment of genetic relationships in *Hippeastrum* using RAPD markers. Plant Biotechnol Rep..

[15] Zhang L, Xu YC, Cheng HZ, Zhou YZ (2012). Genetic relationship analysis and fingerprint construction of 62 cultivars of *Hippeastrum* spp. based on ISSR marker. J Plant Resour Environ..

[16] Tan C (2012). Development of simple sequence repeat markers for bermudagrass from its expressed sequence tag sequences and preexisting sorghum SSR markers. Mol Breeding..

[17] Morgante M, Hanafey M, Powell W (2002). Microsatellites are preferentially associated with nonrepetitive DNA in plant genomes. Nat Genet..

[18] Sathuvalli VR, Mehlenbacher SA (2012). Characterization of American hazelnut (*Corylus americana*) accessions and *Corylus Americana* × *Corylus avellana* hybrids using microsatellite markers. Genet Resour Crop Ev..

[19] Iorizzo M (2011). De novo assembly and characterization of the carrot transcriptome reveals novel genes, new markers, and genetic diversity. BMC Genomics..

[20] Verhaegen D, Fofana IJ, Logossa ZA, Ofori D (2010). What is the genetic origin of teak (*Tectona grandis* L.) introduced in Africa and in Indonesia?. Tree Genet Genomes..

[21] Wang R (2013). De novo sequence assembly and characterization of *lycoris aurea* transcriptome using GS FLX titanium platform of 454 pyrosequencing. Plos One..

[22] He QL (2010). Analysis of floral transcription factors from *Lycoris longituba*. Genomics..

[23] Chang L, Chen J, Xiao Y, Xia Y (2011). De novo characterization of *Lycoris sprengeri* transcriptome using Illumina GA II. Afr J Biotechnol..

[24] Núñez-Acuña G, Gallardo-Escárate C (2013). Identification of immune-related SNPs in the transcriptome of *Mytilus chilensis* through high-throughput sequencing. Fish Shellfish Immun..

[25] Grabherr MG (2011). Full-length transcriptome assembly from RNA-Seq data without a reference genome. Nat Biotechnol..

[26] Verma P, Shah N, Bhatia S (2013). Development of an expressed gene catalogue and molecular markers from the de novo assembly of short sequence reads of the lentil (*Lens culinaris* Medik.) transcriptome. Plant Biotechnol J..

[27] Kanehisa M, Goto S, Kawashima S, Okuno Y, Hattori M (2004). The KEGG resource for deciphering the genome. Nucleic Acids Res..

[28] Jones BC, Womack JE (2012). Polymorphism and haplotype structure in river buffalo (*Bubalus bubalis*) Toll-Like Receptor 5 (TLR5) coding Sequence. Anim Biotechnol..

[29] Wei Z (2017). Assessing genetic diversity and population differentiation of colored calla lily (*Zantedeschia* Hybrid) for an efficient breeding program. Genes..

[30] Wei Z (2016). Transcriptome analysis of colored calla lily (*Zantedeschia rehmannii* Engl.) by Illumina sequencing: *de novo* assembly, annotation and EST-SSR marker development. PeerJ..

[31] Tóth G, Gáspári Z, Jurka J (2000). Microsatellites in different eukaryotic genomes: survey and analysis. Genome Res..

[32] Dutta S (2011). Development of genic-SSR markers by deep transcriptome sequencing in pigeonpea [*Cajanus cajan* (L.) Millspaugh]. BMC Plant Biol..

[33] Wang SF (2012). Transcriptome analysis of the roots at early and late seedling stages using Illumina paired-end sequencing and development of EST-SSR markers in radish. Plant Cell Rep..

[34] Liu S (2011). Generation of genome-scale gene-associated SNPs in catfish for the construction of a high-density SNP array. BMC Genomics.

[35] Vignal A, Milan D, SanCristobal M, Eggen A (2002). A review on SNP and other types of molecular markers and their use in animal genetics. Genet Sel Evol..

[36] Odeny DA, Jayashree B, Gebhardt C, Crouch J (2009). New microsatellite markers for pigeonpea (*Cajanus cajan* (L.) millsp.). BMC Res Notes..

[37] Singh D (2016). Molecular assortment of Lens species with different adaptations to drought conditions using SSR markers. Plos One..

[38] Zhang L, Cheng HZ, Zhou YZ (2011). Research progress of *Hippeastru*m. J Jiangsu Agr Sci..

[39] Yang, L., Zhu, L., Sun, H. M. Research advance of methods about carotenoid analysis in plant. *North Hortic*. 199–201 (2011).

[40] Zhao YQ, L QL (2009). Research advances in the formation mechanism and genetic characters of double flowers. Acta Bot Bore-Occi Sin.

[41] Liu JW, Sun CH, Liu N (2004). The ABC model and the quartet model of floral organ identity. Chinese Bull Bot..

[42] De Hertogh, A., Le Nard, M. Physiology of flower bulbs (Elsevier, 2003).

[43] Arroyo S (1982). The chromosomes of *Hippeastrum*, *Amaryllis* and *Phycella* (Amaryllidaceae). Kew Bull..

[44] Meyer E (2009). Sequencing and de novo analysis of a coral larval transcriptome using 454 GSFlx. BMC Genomics..

[45] Vidotto M (2013). Transcriptome sequencing and de novo annotation of the critically endangered Adriatic sturgeon. BMC Genomics..

[46] Kumar A (2014). Sequencing, de novo assembly and annotation of the Colorado potato beetle, Leptinotarsa decemlineata, transcriptome[J]. Plos One..

[47] Long Y (2015). De novo assembly of transcriptome sequencing in *Caragana korshinskii* Kom. and characterization of EST-SSR markers. Plos One..

[48] Yeh, F. C., Yang, R. C., Boyle, T. J. B., Ye, Z. H. & Mao, J. X. POPGENE, the user-friendly shareware for population genetic analysis. *Mol Biol Biot Cent Univ Alb*e (1997).

[49] Falush D, Stephens M, Pritchard JK (2007). Inference of population structure using multilocus genotype data: dominant markers and null alleles. Mol Ecol Resour..

[50] Gilbert KJ (2012). Recommendations for utilizing and reporting population genetic analyses: the reproducibility of genetic clustering using the program structure. Mol Ecol..

[51] Evanno G, Regnaut S, Goudet J (2005). Detecting the number of clusters of individuals using the software STRUCTURE: a simulation study. Mol Ecol..

