# Author Correction: Revealing the complex genetic structure of cultivated amaryllis (*Hippeastrum hybridum*) using transcriptome-derived microsatellite markers

**DOI:** 10.1038/s41598-020-60510-8

**Published:** 2020-02-20

**Authors:** Yi Wang, Defeng Chen, Xiaofeng He, Jiangxian Shen, Min Xiong, Xian Wang, Di Zhou, Zunzheng Wei

**Affiliations:** 1 Beijing Vegetable Research Center, Beijing Academy of Agriculture and Forestry Sciences, Key Laboratory of Biology and Genetic Improvement of Horticultural Crops, Ministry of Agriculture, Key Laboratory of Urban Agriculture, Ministry of Agriculture, Beijing Engineering Technology Research Center of Functional Floriculture, Beijing, 100097 China; 2 Department of Ornamental Horticulture, China Agricultural University, Beijing, 100193 China; 3 Key Laboratory of Genetics and Breeding in Forest Trees and Ornamental Plants, Ministry of Education, College of Biological Sciences and Technology, Beijing Forestry University, Beijing, 100083 China; 4 School of Basic Medical Science, Guiyang Medical University, Guizhou, 550004 China

Correction to: *Scientific Reports* 10.1038/s41598-018-28809-9, published online 13 July 2018

The Article contains errors.

The Article reports cultivars ‘Chico’ and ‘Jewel’ as double petal. It was brought to the authors’ attention that this information may be incorrect. To confirm the correct assignment, the authors replanted the cultivars used in the study and recorded their phenotypic characteristics again, including flower colour and petal type. These results confirmed that ‘Chico’ is a single petal, ‘Jewel’ is a semi-double petal, and ‘Blushing Bride’ is a single petal cultivar.

Therefore, in the Results subsection ‘The genetic relationships and population structure of cultivated amaryllis’,

“It is also noteworthy that the accessions within each cluster can also be described on the basis of their petal number (single or double) and colour, including ‘Chico’ and ‘Ballerina’ (both with double pink or red flowers) along with ‘Papillo’, ‘La Paz’, ‘Exotic star’, ‘Santiago’, ‘Fairy tale’, and ‘Faro’ (single petals) in cluster I”.

should read:

“It is also noteworthy that the accessions within each cluster can also be described on the basis of their petal number (single or double) and colour, including ‘Ballerina’ (double red flowers) along with ‘Chico’, ‘Papillo’, ‘La Paz’, ‘Exotic star’, ‘Santiago’, ‘Fairy tale’, and ‘Faro’ (single petals) in cluster I”.

and,

“These accessions are distinct from ‘Benfica’ and ‘Cherry Nymph’ (double petals); ‘Bolero’ and ‘Faro’ (single and pink or red flowers); ‘Brazza’ and ‘Adele’ (single and red flowers); and ‘Amigo’, ‘Gervase’, ‘Amorize’, ‘13–47’, ‘Red Lion’, ‘Double Dream’ (single or double and pink or red) in cluster II. All of these varieties are distinct from ‘10–32’ and ‘10–6’ (double, red and white complex colour), which group with ‘Pasadena’, ‘Splash’, ‘Jewel’, ‘Harlequin’, ‘Ice Queen’, ‘Zombie’, ‘Vegas’, ‘Ragtime’, and ‘First Love’ (double peals) in cluster III.”

should read:

“These accessions are distinct from ‘Benfica’ and ‘Cherry Nymph’ (double petals); ‘Bolero’ and ‘Faro’ (single and pink or red flowers); ‘Brazza’ and ‘Adele’ (single and red flowers); and ‘Amigo’, ‘Gervase’, ‘Amorice’, ‘13–47’, ‘Red Lion’, ‘Double Dream’ (single or double and pink or red) in cluster II. All of these varieties are distinct from ‘10–32’ and ‘10–6’ (double, red and white complex colour), which group with ‘Pasadena’, ‘Splash’, ‘Jewel’, ‘Harlequin’, ‘Ice Queen’, ‘Zombie’, ‘Vegas’, ‘Ragtime’, and ‘First Love’ (semi-double or double peals) in cluster III.”

In addition, in the Discussion subsection ‘Cluster analysis and genetic structure within Hippeastrum spp. accessions’,

“However, the N-J tree and structural plot generated in this study (Fig. 4) demonstrate that the 104 accessions we considered cannot be fully clustered according to the number of flowers or their colour. Exceptions include the ‘Lemon sorbet’, ‘Ballerina’ and ‘Chico’ varieties. Of these varieties, the former possesses green single petals, whereas ‘Ballerina’ (red flowers) and ‘Chico’ (pink flowers) both have double petals. These exceptions are important because they illustrate the comprehensive nature of amaryllis population structure.”

should read:

“However, the N-J tree and structural plot generated in this study (Fig. 4) demonstrate that the 104 accessions we considered cannot be fully clustered according to the number of flowers or their colour, which illustrate the comprehensive nature of amaryllis population structure.”

Finally, the corrected section of Supplementary Table 5, listing the characteristics of the affected cultivars, is included below as Table 1.

**Table 1:** .

Cultivars/hybrids	Origins	Spathe Color	Type
Chico	Netherlands	pink	single petal
Jewel	Netherlands	white	semi-double petal
Blushing Bride	South Africa	pink	single petal

There are also several typographical errors in some of the names. Cultivar ‘Amorize’ should be ‘Amorice’, and *H. solandrifoliu* should be *H. solandrifolium*.

These changes do not affect the overall conclusions of the Article.

