# Supplementary material for: Revealing the complex genetic structure of cultivated amaryllis (Hippeastrum hybridum) using transcriptome-derived microsatellite markers
Source: Sci Rep. 2018 Jul 13;8:10645. doi: 10.1038/s41598-018-28809-9 (PMC6045658; doi:10.1038/s41598-018-28809-9)
Supplement: Supplementary file 4 — LaTeX Supplementary File [file 41598_2018_28809_MOESM4_ESM.pdf]

Revealing the complex genetic structure of cultivated amaryllis (*Hippeastrum hybridum*) using transcriptome-derived microsatellite markers

Yi Wang [1,2,3,†], Defeng Chen[1], Xiaofeng He[1,4], JiangXian Shen[1,4], Min Xiong [1], Xian Wang [1], Di Zhou [1,*], Zunzheng Wei [1,*]

[1] Beijing Vegetable Research Center, Beijing Academy of Agriculture and Forestry Sciences; Key Laboratory of Biology and Genetic Improvement of Horticultural Crops, Ministry of Agriculture; Key Laboratory of Urban Agriculture, Ministry of Agriculture; Beijing Engineering Technology Research Center of Functional Floriculture, Beijing 100097, China;

[2] College of Horticulture Science and Technology, Hebei Normal University of Science &Technology, Qinhuangdao 066600, China;

[3] Key Laboratory of Genetics and Breeding in Forest Trees and Ornamental Plants, Ministry of Education, College of Biological Sciences and Technology, Beijing Forestry University, Beijing 100083, China;

[4] School of Basic Medical Science, Guiyang Medical University, Guizhou 550004, China;

[*] Correspondence: zhoudibvrc@163.com, weizunzheng@163.com

[†] These authors contributed equally to this work.

Table S4. Primer sequences of 335 Hippeastrum SSRs used for marker validation.

Locus	Unigenes	Motif	Forward primer (5'-3')	Reverse primer (5'-3')	Size (bp)	Ta
FP001	comp100051_c0_seq2	(AATT)5	CACGGAGTAGCTGATGTTGC	CACCCCCAAGTAGAGACGAA	238	59.8
FP002	comp100209_c0_seq1	(TTC)8	CGTAGCGAAGACGAGGAATC	TTTGTTGTGCTCTGAAACCG	200	59.9
FP003	comp100588_c0_seq2	(AAT)7	ACATGAGCAAACGCCATACA	TCGCTACTTGGAAGTTTTTGC	157	59.8
FP004	comp100681_c0_seq7	(GAG)7	CGACAGCCATCTGTTGCTTA	TAAAAACCCTAACCCTGGCG	126	60.6
FP005	comp100832_c0_seq2	(GAA)7	ATTTGGCGACGAAGATTACG	ATTCCATTAATGTCGCCAGC	255	60.0
FP006	comp100832_c0_seq2	(ACA)7	CCCAACTACCCGAAAGTCAA	CGTAATCTTCGTCGCCAAAT	262	60.0
FP007	comp100879_c0_seq2	(TTTA)5	AATGTGATCCAAAAGCCGAC	TTTGAGGGTAAAAGTAAATGCCA	142	59.9
FP008	comp101051_c0_seq1	(CT)6aacctca(CT)6	CTTTCCCTCCACATTTCCCT	GATCCGGGTTCCTTTTCATT	271	60.2
FP009	comp101269_c0_seq1	(TG)11	CACACATGCAACACCAACAA	GCCCCAACTCAGAACAGAAA	226	60.1
FP010	comp101298_c0_seq3	(CATG)6	ACTCGGTTTCGATTTCGCTA	TTGATTAAGTTTGCTAAATGGTTGA	170	59.5
FP011	comp101612_c2_seq1	(ATTT)5	GAGGCCCTCAAATGTTTCAC	ATGCACGGAGGTACACATGA	157	59.8

FP012	comp101645_c0_seq2	(CT)6----(CT)9	TCAAACTCTCACTCTCCCAGC	ATTGACGGCAAGAGAGAACG	161	60.0
FP013	comp101792_c0_seq1	(ACA)7	TCATGACACCTTCGGAGACA	CCAAATGATCCCAAGGAAGA	187	60.1
FP014	comp101799_c0_seq1	(GA)8(GC)6	CATTTGGCATTGCATGGTAG	TATTTTATGGCCAGGTTGCG	226	60.4
FP015	comp102019_c0_seq9	(TCA)7	TCCGATCCATAGCCACTTTC	TCCCATGGTCTTATCCTTGC	227	60.0
FP016	comp102443_c0_seq2	(GA)10	GTGAGCTGTCGATGGGAGTT	GTCGACCGCCACCTACTG	228	60.3
FP017	comp102528_c0_seq3	(CG)6agagc(GA)6	GCAGATGTTGAGGAAGAGGC	CCCGGGTATTCAACGATAAG	194	59.6
FP018	comp102931_c0_seq2	(CT)8cccca(TG)7	CCCTGGGCATCATCTAGAAA	GGCAATCTGAAGCACATCAA	247	59.9
FP019	comp102951_c0_seq1	(CTG)7	AGTAGCGACGGCAGTTCTTG	TCGTTTCACACCATCGAAGA	160	60.4
FP020	comp103335_c0_seq2	(CT)10	ATACCCATTCCGAATCAACG	TGAGCTTGAAGAGGGAGGAG	195	59.7
FP021	comp103500_c0_seq1	(CGC)7	AGGGTTAGGGTTTGGATTGG	TTTTACATTTCCACCCGTCC	168	59.9
FP022	comp103613_c1_seq2	(GAGC)5	GGCAAAGAACCCATTTCCTT	GAGCGTCGTCTTTAGGATGC	178	60.1
FP023	comp103675_c0_seq3	(TTC)7	AACTGAGCTGCCTCTCTTGC	AAGAAGTGGACTGACACGGG	113	60.0
FP024	comp103878_c0_seq1	(TCC)7	TCACAGATTCTGGATGAGATCAA	TCTGTGGTTCTTTTGCGATG	100	59.8

FP025	comp103999_c0_seq2	(CA)11	GCAGGCTACAATCACATCCA	AATGGCAAAATGCTGAAAGG	110	59.9
FP026	comp104029_c0_seq2	(AC)11(TC)7	ATGTTGCTGCACTTGTCCAT	TCTTTGCATAATTCTCGGCA	265	59.3
FP027	comp104487_c0_seq1	(TGT)7	CCAAATGATCCCAAGGAAGA	TCATGACACCTTCGGAGACA	187	60.1
FP028	comp104775_c0_seq1	(AGA)7	AGGTCACTTTCACCAAACGC	ACCCACACGCATTATGACAA	273	60.0
FP029	comp105188_c0_seq1	(CGG)7	TTTTACATTTCCACCCGTCC	AGGGTTAGGGTTTGGATTGG	168	59.9
FP030	comp105210_c0_seq2	(AT)11	TGCCCACTGGGAACAAATTA	CAGGACCACACAAAGCTCAA	257	60.6
FP031	comp105239_c0_seq5	(CCT)7	ACCCCTTTCCTTGCCTTCTA	GCGTTAGCCAGGGATTTTAG	232	59.5
FP032	comp105283_c0_seq3	(ATG)7	TACGGAAACCAAGGATGGAG	ACATTAGCATCTCGCACACG	227	59.9
FP033	comp105616_c0_seq1	(GAG)7	GAGTCGAGGTGGGTGTTGAT	ATCTCCCCAAACCCCATAAG	127	60.0
FP034	comp105647_c0_seq1	(CT)7---(CT)6	TCTCATAAAAGCTACGAAGGGC	GGCGAAATCCTAGATCACCA	125	60.0
FP035	comp105812_c0_seq1	(AAG)8	ATAACAACAAACGGGGCAAA	AATCCCTTGAGCTGAGAGCA	233	60.2
FP036	comp106041_c0_seq2	(CAT)9	ACGGTTCACATTCCATCACA	GCAAACGAATCTGTCTGCAA	204	59.9
FP037	comp106076_c0_seq1	(AT)11	ATTAAGCCATCACTGGCGAC	ATACTTCCTGGTTCCCGAGG	224	60.2

FP038	comp106408_c0_seq1	(TGA)7	TGCTCTTTGGGCTTCTTCTC	GCCCTAAACCCCTCAACTTC	130	59.8
FP039	comp106996_c0_seq3	(TCA)7	GCCCTAAACCCCTCAACTTC	TGCTCTTTGGGCTTCTTCTC	130	59.8
FP040	comp107304_c1_seq2	(GA)6gg(A)10	GAAAACCGCAATGAGAAAGC	ATGGAGTCCTTCAACAAGCG	275	60.0
FP041	comp107699_c0_seq2	(AATC)5	CCAACCTGACCCATTGTCAT	TGGTGATGCTAGTCAGGTGC	203	60.2
FP042	comp10776_c0_seq1	(CA)6--(CA)6	TGTTTATTGTGCAGGGCAAG	AAGCATCGAAACCTGAAATGA	279	59.7
FP043	comp108017_c0_seq3	(TTTA)5	AGTCTCGCCCAATCAGTTGT	GTAAGAAGGAGATGCTGCCG	188	59.9
FP044	comp10870_c0_seq2	(ATA)7	CTCTGTTGGGTGGAAGTTGA	CATCGTCACCACTCCTTCCT	146	59.4
FP045	comp108875_c0_seq1	(CGG)7	GGCCAAATCAAGAAACCTCA	ATGTCTTTGTCGTTCTCCGC	259	60.2
FP046	comp110005_c0_seq1	(GA)10	GTGAGTGAGGAACAGGGAGG	TTTTGGGCATGTAATCGTCA	266	59.8
FP047	comp110008_c0_seq1	(AGA)7	TGAATGTGTTTGAGGCTTGC	TTGATCCTCTTTTCTTGTGGC	177	59.6
FP048	comp110594_c0_seq2	(TTTA)5	TTTTCTTCTCCAGGGTGGTG	CCCCGCATTTTCCTTTATTT	194	60.1
FP049	comp111096_c0_seq1	(GCG)7	CCTTCACCAGCTTCTCCAAG	CTCTCCTCGCTCACATCCTC	187	60.0
FP050	comp111309_c0_seq1	(TGA)7	TCCCATGGTCTTATCCTTGC	TCCGATCCATAGCCACTTTC	227	60.0

FP051	comp111543_c0_seq1	(GA)6(GT)6	AAACTTGGGACTTGGAGCG	CTACAACGCCTGTTGAAGCC	125	60.5
FP052	comp111562_c0_seq1	(ATG)7	GAAGTCGAAGTCCAATCCCA	CTGTCATCCATTTCTGGCCT	276	60.1
FP053	comp111578_c0_seq1	(GGA)7	TCTTCTACAACCAAACCGGC	TGGCCTGACCATTGACATTA	253	60.0
FP054	comp111586_c0_seq8	(CGT)7	ATCGTCTATCCGTTGCCATC	AGGCGGAAGTGAAGAGCATA	166	60.0
FP055	comp111657_c0_seq2	(GA)10c(AG)8	TTCCCAGTCTCCGATTCAAC	TTTTCCTTCTTTCTCTTTCTTTTCC	244	59.9
FP056	comp111771_c0_seq1	(TCA)8	ACTCACATCGCGAAGCTTTT	GCACCGAGTGTGGTTAGGAT	122	60.0
FP057	comp111942_c0_seq7	(TA)7tt(TG)7(CG)8	ACAGAGGAAGTGTGGGAAGC	ATGTCGTCATTGCCAACAAA	147	59.6
FP058	comp112199_c0_seq5	(TTAT)5	AGCGGGCTCCACTTTTTATC	AGGAGGCTTACTCGGGTCAT	272	60.3
FP059	comp112479_c0_seq1	(CGC)7	CTCTCCTCGCTCACATCCTC	CCTTCACCAGCTTCTCCAAG	187	60.0
FP060	comp112651_c0_seq1	(AGG)7	GTTATTTCGAGCAGCGGAAG	CGAGTCAGCAGCAAGATCAA	212	60.1
FP061	comp112722_c0_seq1	(GGCG)5	TGCAATGACTCATGCCAACT	TACTCATGGGGGCTGATTTC	214	60.1
FP062	comp1131_c0_seq1	(TTTA)5	GTTGATCGTGGGGAAATCAT	TGGAGGCTCAGTATCATTATTCAA	210	59.8
FP063	comp11314_c0_seq1	(TG)10	GCTGCTGCAGAAAGAACGTA	AGGGCATGAGGTAAAACTGG	193	59.2

FP064	comp113156_c0_seq1	(TTC)7	TACGTTCGATTTAGGACCCG	GGAACATGAAGGTTCTTGCC	255	59.7
FP065	comp113187_c0_seq2	(CT)10	TCCGTCCATCTATACTCGGC	GGAAGATGTGATACCTGCCC	226	59.7
FP066	comp113277_c0_seq3	(TA)11	GGGTTTCTACACCATCGCAC	TAGTCCTGGGGATCATCCTG	230	60.1
FP067	comp113434_c0_seq3	(CAG)7	AGGTTTTCCCGAGCTGATTT	CTGGTTTGCGGGACTATGTT	230	60.0
FP068	comp113640_c0_seq6	(AATA)5	CTGAGAGAGGTGCCAACAAA	CCATTTGGACGTCAAACAACT	244	59.4
FP069	comp113793_c0_seq1	(TCT)7	TGGCTGGGAATAATTAACGC	CCCAGGAAAAATGAACTTGC	211	59.7
FP070	comp113908_c0_seq8	(TGA)7	CCAAGGAGTACGAGGACCAA	GGAAGCGCTGAATCATTCTC	170	60.0
FP071	comp114140_c0_seq2	(TCC)7	TGGCCTGACCATTGACATTA	TCTTCTACAACCAAACCGGC	253	60.0
FP072	comp114300_c0_seq1	(CTTT)5	TGAATTCCAATGGTGCTGAA	CCAAATCAAATGAGGGGAGA	207	60.0
FP073	comp114342_c0_seq6	(AAG)7	AAAAGCCTGTGGCAGAGAAA	GTGGACCTGCTTCAACACCT	209	60.1
FP074	comp114742_c0_seq1	(CT)8cattgttggtt(TC)8	GATCCCCTCTCTTCCTCGAT	AGAGAAAAGGGTGGGGAAAA	175	59.8
FP075	comp114764_c0_seq1	(CT)6---(TC)6	TCCCTCTACCTCTGGCGATA	TCACCTGTTCACCAGGGTTT	259	60.1
FP076	comp114898_c0_seq6	(AGA)7	GCATCCTTTTGGCATTCTGT	CTCCTCCCCTTCTTCGTCTT	236	59.9

FP077	comp114987_c0_seq7	(CCCT)5	GGCATTATCACGCCTAAGGA	CACCACAAGAAACCGAACAA	203	59.8
FP078	comp115192_c0_seq3	(TTA)7	GTTCCAGCGGTGTTTGAAAT	CAACGACGATAGCAGAAGCA	167	60.1
FP079	comp115295_c1_seq8	(AT)7--(AT)7--(AT)7	AAGCGCCATTGCCTATTCTA	CCTGATCAAACGGGACAAGT	200	59.9
FP080	comp116188_c0_seq4	(TC)6--(CA)7	CCAAACCTCGAACACCTCAT	GTGGAGAGTGTGAGGAGGGA	248	60.1
FP081	comp116297_c0_seq3	(AAGA)6	GTTTTCCTTGTTCTGCGAGC	TTGAATCATTGACTGGCTGC	276	59.9
FP082	comp116351_c0_seq1	(GGA)7	GTTGCGATTGGGTTTGATTT	AAACCCTTCTTCCCCTGGTA	167	59.8
FP083	comp116381_c1_seq4	(TTTA)5	CCAACTGTAAGAAACCCCCA	CCCAAAGGCCTAAATTCACA	191	59.9
FP084	comp116522_c0_seq3	(TCC)8	CCACCCATCTCCTACCCTTT	CTGGTGGAGGTTCCCTATGA	135	60.1
FP085	comp116547_c0_seq1	(GCA)8	CCGAGATGGCAGGTGTAAAT	CTATGTGGTGGAGGAGGCAT	187	60.0
FP086	comp116574_c0_seq7	(CA)10t(AC)8	GCCCCAACTCAGAACAGAAA	AAACAATTCAAATGGGCGAG	253	60.1
FP087	comp116772_c0_seq2	(TA)11	TACTGCCATTTGACTGAGCG	CAGCAGACCAACTGTGGCTA	250	60.0
FP088	comp117059_c0_seq2	(TA)7---(CT)6	GTGTGGTCCATAACCTCCGT	AGAAGTGGCGACTCCAAAGA	195	59.8
FP089	comp117417_c0_seq1	(GGAG)5	GAGGATGCACTCTTTGAGCC	CGTCAACTCCTCTTCCTTCG	212	60.0

FP090	comp11767_c0_seq1	(TC)8---(TC)6	CACATTCCGAGTGCAGTTTG	GTGCCATCGTTTTCCTTGTC	195	60.4
FP091	comp117821_c0_seq1	(CAG)7	ATCCAATGCAACAACTGCAA	CCTCCATTTCCTCGTTTCAA	152	60.1
FP092	comp117837_c0_seq3	(GCT)7---(TGC)6	ATAAAGCGGGGGAAGATGAT	TCCTGCACCACCTACCTACC	193	59.9
FP093	comp117951_c2_seq15	(CA)12	TCCAAACATTTTACCCCCAA	ATACTTGTGAGGGGGAGGCT	183	60.0
FP094	comp118506_c1_seq1	(ATGG)5	GGAGATCCTCATCTGGGTCA	CTGATCTGCTCGACAACAGC	273	59.9
FP095	comp118616_c0_seq5	(GA)10	CATCCCATTGGACAACAAAA	GTCCGCTGACATCTGAGACA	246	59.6
FP096	comp118639_c0_seq3	(CTC)7	TTCCCTACCCTATCTGCCCT	AGGAAGTTTTGGCTCGATGA	234	59.9
FP097	comp118757_c0_seq1	(GCA)6----(CAG)7	TCCTGCACCACCTACCTACC	ATAAAGCGGGGGAAGATGAT	193	59.9
FP098	comp118864_c0_seq1	(TTTC)6	CTGAGAGGGCCAATTCAGAG	GCAATGCAACCAGAACACAC	275	60.1
FP099	comp118925_c1_seq6	(ATTT)5	TGCGTCGGTTGTACATTGTT	AGGGCATGGTCAGTTGGTAG	240	60.0
FP100	comp118964_c0_seq1	(ATGA)5	CCTCGAGAGGCTCACGTATC	TCCCATCAATGCATACTTTCTC	256	59.5
FP101	comp119001_c0_seq7	(CAG)7	TGCCAGCCTTGATTCTCTTT	GGAAAGTGGAACGGAGACAA	203	60.0
FP102	comp119266_c0_seq6	(TTC)9	TACTTCTTCATCATCGGGGG	CCACTCCGTTTTGCTCTCTC	215	59.9

FP103	comp119437_c1_seq2	(ATTT)5	GCAAGATTGGATCTCCATCAG	GCTCCAACCTGGCATTAAAA	137	59.9
FP104	comp119628_c0_seq2	(C)12---(TC)10	TCAACATCAAGCAGTTCCCTT	AAATCCAATGGGGTGGAAAT	182	60.0
FP105	comp119667_c0_seq1	(GAA)7	CGGTGGGAGAAGAAGAGATG	GAGACGATGAAGCTCCGAAT	199	59.6
FP106	comp119748_c0_seq2	(GAC)8	GAGGTGAAGATCTCATCGGC	GCTGCTCAGCTGAAACCATT	126	60.2
FP107	comp120080_c1_seq1	(TTC)7	GATTGTCGAACAATGGGCTT	GAAGCAGTGAGTAACTCTGACTAATTT	130	59.0
FP108	comp120140_c1_seq2	(AGA)7g(GAA)5	TCTTTGGGGTTCGAATTGAG	GCAGGATCTGCAACCTTCTC	264	60.0
FP109	comp120323_c0_seq6	(ACC)7	ACAAAAATGGCCATCGTCTC	TGATGAGGAGGATGAGGAGG	161	60.0
FP110	comp120361_c0_seq6	(GGA)7	GCAACCTCAACACCAAAGGT	TACCAGCAACAGCACTACGG	241	60.0
FP111	comp120395_c0_seq7	(AG)10	TTTGCAGTTGGCATGTTGTT	AACCCCGAAGAGACGAGAAT	276	60.1
FP112	comp120522_c1_seq4	(ATAA)5	GACTTTGGTTCGGGTTTTGA	ACGAAGGAGAGGATTTGGGT	210	59.9
FP113	comp120562_c0_seq9	(CAG)7	ACTCTACCTCCGAGATCGCC	AATCATTCAAGTTGGGCAGC	263	60.4
FP114	comp120590_c0_seq4	(TTTG)5	GATTCCACCACCAAGGATTG	CTCAACCTCAATTCCCCAGA	270	60.1
FP115	comp120638_c0_seq3	(TA)10	CGGGTCAATGTTAAGCCAGT	CAGGTGATGAGCATTGGATG	151	60.0

FP116	comp120723_c0_seq2	(AG)7---(AG)7	TCGGGGCAGACATCTTTAAC	GCTTTGGGAGGTATTTTTGTGA	253	60.2
FP117	comp121003_c0_seq1	(GGT)7	AACTTCAGCACTCGCGAAAT	TAAGCCTCCAAAAGAACCGA	193	59.9
FP118	comp121111_c0_seq1	(CT)8---(TC)9	TCCTAGGGCTTCGTCGAGTA	CCATTTTTCCAAAACTCGCT	262	59.6
FP119	comp121123_c0_seq9	(CCG)8	CTCATTCGTCGAGTCCACCT	GGATATCCGTCGTACATGGC	183	60.2
FP120	comp121193_c1_seq1	(CT)6---(TC)6--(TC)6	TACCGAACCATAGCCGTCTC	GAAATCAAGGCATCCTTCCA	249	60.1
FP121	comp121278_c0_seq8	(AATG)5	CGATCTCGGCTATGTGAATG	TGCATGAGGGGTAATCACAA	169	59.6
FP122	comp121287_c1_seq5	(TC)10	AACCCCGAAGAGACGAGAAT	TTTGCAGTTGGCATGTTGTT	276	60.1
FP123	comp121337_c1_seq3	(TCC)8	ACAGCACTACGGCACCTTCT	CCTCCTCCTCTTCGTCTCCT	249	59.9
FP124	comp121466_c0_seq5	(CTTC)5	GGTTTTTGTTCGGGAGTGAA	TCTTCCCATTGGACGTTCTC	214	60.0
FP125	comp121583_c0_seq46	(TG)8tacatatta(TG)6	CAGCTACTATTTTATTTATCGGCCA	TTTGCCATCATCCAGATTCTC	247	60.0
FP126	comp121745_c0_seq1	(AT)11	GCGTTTGAAGCTGATGTTGA	AGAGGGGAAAGAGAAAGGGA	125	59.6
FP127	comp121764_c0_seq6	(TG)11	TATACCACCCCTGCAGGAAC	TCCTTCAGCCATAAAAGGGA	258	59.7
FP128	comp121921_c0_seq7	(TAT)7	TGGGAAGGCAGTGAGTTTTC	TCGGTTTGCTCTGTCAGTTG	184	60.1

FP129	comp122097_c1_seq6	(AGA)8(TGA)5	GCTTCAGAGAAGGATGCAGG	TGGCGGTTTTCTTCTTCTTG	187	60.2
FP130	comp122110_c0_seq13	(AC)10	CACCAATTGAAAACCAAGGG	CCAGAGATGAAACAAGGGGA	272	60.1
FP131	comp122222_c1_seq4	(AG)10	TCGAGGTGCTGTTTGTTTTG	AGACCAACGCAAGTCAGTCC	125	60.1
FP132	comp122442_c0_seq7	(CGA)8	TAATTGCCAACTCTCGTCCC	CATGCACCATACATGCAACA	226	60.0
FP133	comp122453_c0_seq2	(CTG)7	CCTCCATTTCCTCGTTTCAA	CAGATGCAACTGAACCCTCA	177	59.9
FP134	comp122537_c0_seq1	(AAG)7	TAGCCATGGGAGTAGGAGGA	GCAATTTTGACTAAGGCCCA	198	59.9
FP135	comp122710_c1_seq1	(TC)10	AAATGAGTTGGTGCTGACCC	AGCATGTGTCGTTTACGTGC	247	59.9
FP136	comp122812_c1_seq16	(GAG)8	GAGCTTGACCTGACGGACTC	GCAGAGCATGGCTTCTATCC	190	60.0
FP137	comp123324_c0_seq1	(TC)6cg(TC)8	GCAACGGTCTTGAAGTGGAT	TGCTCTCCTCTTTCTCTAACACAA	250	59.9
FP138	comp123377_c0_seq85	(CA)10	GCTTGTAGGCTTCAAATCTGC	TCAACTTGAACATGCTCGGA	233	59.8
FP139	comp123592_c0_seq11	(TGC)7	CCATGGATAGAACACCACCC	GCCGAAGTAGAAGCTGAACG	200	60.1
FP140	comp123657_c0_seq15	(ATC)8	AGCTGCCATGGCTCTTAGAA	TTGCTTGGTGGTGTGACAAT	232	60.1
FP141	comp123729_c0_seq1	(TTTA)5	TGGAGCGAAATAGGAAATGC	AGTTGGCAAATGCCGTAATG	210	60.5

FP142	comp123761_c0_seq7	(CGG)7	CTGCAGCAGCAGCTCACT	CACTGATGAATTGCCTGCAT	210	59.4
FP143	comp123851_c0_seq12	(TG)10	AAGAACCCGTGATGACTTCG	TTTTGGATGCACATACTGCC	219	59.8
FP144	comp123991_c0_seq1	(TGG)8	GGAAAAGATGACGGTGGAGA	CACTCTCCCCAAAAATCTGC	262	59.9
FP145	comp124169_c0_seq1	(GTG)8	GGTCGTCGTCATTGGAATTT	AGCAATGCAAAGCTGATCCT	244	59.9
FP146	comp124259_c0_seq1	(CGGG)5	CAAAATCCCCACATGCATAA	ATTGTTTGGGGAAGCAACTG	240	59.6
FP147	comp124265_c0_seq1	(TA)11	AACCTAGAGCTTGCAACGGA	AGCGACATCGGTAGCATCTT	261	59.9
FP148	comp124393_c0_seq1	(AGC)7	TGAGGAGATGCAATCGTCTG	CCTCGCTTTGAAAACTGCTC	258	60.0
FP149	comp124478_c0_seq2	(TTTG)5	TCATTGCACCAGTTCCGTTA	ATGGCACGAGTCTTTTCCAG	130	60.2
FP150	comp124601_c0_seq2	(GAA)7	CGACTTCTTTGAAACCCTCG	ATCTTGCTCTCCACTGCCAT	233	59.8
FP151	comp124688_c0_seq1	(GA)10	AGCATGTGTCGTTTACGTGC	AGAGTGTTGGTGCTGCCTCT	198	59.9
FP152	comp124694_c0_seq12	(AGA)7	TCACCCCCGAAGATGATTTA	TGTCCACAAACGCATTGATT	221	60.1
FP153	comp124744_c0_seq25	(CT)10	GACGGGTAGCCCTATTCTCC	TTCTGGCTCGTCCTCAGATT	250	59.9
FP154	comp124771_c0_seq14	(TCT)7	GGGGTGGGTGCCTATTATCT	TTTCATGAAGCCACCATTCA	238	60.0

FP155	comp124892_c0_seq1	(CTT)7	ATTGGAGCGAAATTAGGGCT	ATGATTCCCCAGCTCATGTT	258	59.7
FP156	comp125177_c0_seq1	(GAT)7	TCCACTTGATAATCCTCCGC	AGGGTCTCCATTGGGTAAGG	189	60.1
FP157	comp125237_c0_seq2	(GAA)8	CCACTCCGTTTTGCTCTCTC	TCATCAACAACGTAGCCAGC	137	59.9
FP158	comp125266_c0_seq2	(CAC)7	CTCCGATCTATCCCCAACAA	ATTAGAGTTTGTGGTGGCGG	235	59.9
FP159	comp125573_c0_seq2	(CCA)10	GCCACCACCTGCTAGTTCTC	AAAGATGACGGTGGAGATGG	267	59.9
FP160	comp125885_c0_seq1	(GT)10	TCCGGTTCATCATCTTCCTC	ATCCTAGAACAACCCCACCC	218	60.0
FP161	comp125931_c0_seq2	(CT)10	CTCCCTCATCCTCCCTCTCT	AGGGTTGAAATTTGGGGAGA	214	60.2
FP162	comp126273_c0_seq4	(AC)10	AGCCAAAACCCAATTGTTTG	AATGTGATCCAAAAGCCGAC	177	59.9
FP163	comp126275_c0_seq9	(AC)10	GGGTACCCAATTCGAAACAG	TTTTTGCCCTTCTTAACTTCTCTC	112	59.4
FP164	comp126306_c0_seq2	(AC)7---(AT)6	ACATTTTGGCACGTTCACAC	TGTGATTGGAGGAGGGTAGG	214	59.7
FP165	comp126543_c0_seq1	(ATT)8	AGAGAAGGGGCACTGACTGA	TTTTTCTTCCGACTTCATTTTTC	170	59.4
FP166	comp126549_c0_seq4	(CAGC)5	ATCTGGTCACCACGGAAAAC	CCTCCTAATCATGGCTTCCA	187	59.9
FP167	comp126562_c1_seq8	(CGC)8	CAGCATCGACACAAAAGGAA	AGGTGGAGGAGGAAGAGGAG	232	59.8

FP168	comp126685_c0_seq21	(GCG)8	AGGTGGAGGAGGAAGAGGAG	CGATAACATCCCTCCCAATG	271	60.0
FP169	comp126706_c0_seq6	(TCC)7	AAGATCAACGTCCACTTCCG	TCTTGTAACACCCCTCTGGG	153	60.0
FP170	comp126775_c0_seq3	(TAA)8	TCGTCAATGAAAAGGGTAGACT	CTTTTGCGTGGGAATATGGT	152	58.8
FP171	comp126861_c0_seq8	(GAT)7	GGCCAAAAAGTCAACCTTCA	CTCCGTAGCACTCTGGGAAG	273	60.0
FP172	comp126868_c0_seq1	(CAG)7	GCAAGATCCAATTTCCTCGT	CAAAACTTGGTGTTGGTGGA	114	59.3
FP173	comp126883_c0_seq7	(GATC)5	ATCGATCACCGTCAAAGTCC	GAAGCGATCCATTTCTTCCTT	268	59.8
FP174	comp127112_c0_seq8	(AGC)8	GGCTCTTGTACCAAACTCGC	TATGCTGATGAGGCTGATGC	107	59.9
FP175	comp127181_c0_seq3	(TA)11(TG)8--(CA)6	AAAATGAACCTGTATCGCTGG	GTTTGCCGTGGACAAAGAGT	253	59.6
FP176	comp127208_c0_seq2	(AGC)8	AATCTCCCCAATGGAAGACC	GAAGTGGTAACCCCAAACGA	196	60.0
FP177	comp13124_c0_seq1	(AC)10	CTTCCGGTACTTTGTTGGGT	TGAACGAAGGATTGAGAATGG	217	59.5
FP178	comp131564_c0_seq1	(ATTT)5	ACCTCTTCATCATCGCCATC	GTTAGGGTGTGGGGGAGAAT	228	60.0
FP179	comp133852_c0_seq1	(TATT)5	AAAGGGACATGTGCACCAAT	TCCTTCTGGGGAGAAAAGAAA	280	60.2
FP180	comp13476_c0_seq1	(AT)10	CGCGAAATGAATAGGCAAAT	AGCCTGACATGAACGGAGTT	226	59.9

FP181	comp137584_c0_seq1	(ATTT)5	ACATGGGGGTTTCGGTAAAT	CCAGCAGTTTGGTGGAATCT	274	60.2
FP182	comp137748_c0_seq1	(A)10gg(AC)8	TACCACTGTGTCAGACCCCA	CCATAACCCCAACATCTGCT	262	59.9
FP183	comp137844_c0_seq1	(TTTA)5	GACAAGTACCTCAAATTTGCTCG	TTGGACCAACCAAGGATAGG	107	60.0
FP184	comp139013_c0_seq1	(TTTG)5	AATCTCTGCATGTCCTTGCC	ACAAAACACCCACACCCACT	148	60.2
FP185	comp139134_c0_seq1	(TC)10	CAACCGACCTTGCTCAGATT	GGGAAGGAGAGATCGGAAAC	183	60.1
FP186	comp13941_c0_seq1	(TTAA)5	TTCAATCACGTGCTTAATTAGCTT	TTGAAAAACCCATGTGATGC	101	59.4
FP187	comp140330_c0_seq1	(AC)7---(AC)9	GGAAAGGTCCGAGACATGAA	CCTAGAGAGCAACTTAACCGC	105	59.2
FP188	comp140405_c0_seq1	(CAT)7	TTGCATGATGTTTTCGTGGT	GCTGAGAAAGCAGCATTTGA	136	59.6
FP189	comp142393_c0_seq1	(TC)6ccc(CT)7	ACTCGCTTCGTTCCCTCTCT	GAGATCGGTTGCTGGATGAG	104	60.7
FP190	comp143814_c0_seq1	(GAA)7	CTTGATAATGGGGTCACGCT	ACCATGGCCTTAAAGAACCC	108	60.1
FP191	comp147845_c0_seq1	(AT)7agacatgttc(GT)7	CGCACACGTTCACATACACA	CCCCTCCTTATACATACACACACA	258	60.1
FP192	comp148100_c0_seq1	(TC)8cgtgtgt(GC)6	AATCGCTACCCCTCCCTTC	GAGGGATGGAAATCACGCTA	179	60.2
FP193	comp150722_c0_seq1	(TG)6---(TG)6	AAGCATCGAAACCTGAAATGA	TGTTTATTGTGCAGGGCAAG	279	59.7

FP194	comp150966_c0_seq1	(TC)6---(AG)7	GCTGTTATTTCGACCCCTCA	CCCCATTTTTCTTCTCCCAT	116	60.1
FP195	comp153897_c0_seq1	(GA)10	AGTGATGCCTTTTGGTTTGG	CAAAAATCCTCGCCTTTTGA	132	60.1
FP196	comp153925_c0_seq1	(ATTT)5	GCGATGTTTCTGGGTCTTGT	GATACTTGTAAACGTAACCAAGATTGT	126	59.2
FP197	comp154328_c0_seq1	(AC)7agat(AC)8	AATTCCATCCTACCCAACCC	TGGTATAACCTATGTGCCCG	183	59.1
FP198	comp155293_c0_seq1	(TATT)5	TGATGGTTACGAGTTCAAAGCA	GGAGCAACCCATACGAGAAA	106	60.4
FP199	comp155945_c0_seq1	(CATA)5	TTCTTGGTTACACCCAGGAAGT	TTATGGGCGTTTGTTGGAAT	120	60.0
FP200	comp156061_c0_seq1	(CCAT)6	TCCACTCATCCACTCATCCA	GGCTGAGATGAACTAGCCATT	145	59.2
FP201	comp156893_c0_seq1	(TA)10	TTTTTGGGGCTGTTTTTGAG	TATTCCATTTTACACGCGCA	168	60.1
FP202	comp160442_c0_seq1	(CT)7cca(T)10	TTTTACAGGCCCCCTCTCTT	AATAGTGCCCAGGCCTTGTA	163	59.8
FP203	comp160568_c0_seq1	(CCCA)5	CCCATAATTGATGGTGGCA	GAAAAGAAAAGAAAATGGGAAAAA	220	59.6
FP204	comp16115_c0_seq1	(AGA)7	TTGAACCAGATCAATGTCGC	CTCCACTTTCAAGATCCCCA	241	59.8
FP205	comp161324_c0_seq1	(CG)7---(CG)7	GCCACAGCAGATTCCAGAAC	CAGACGTGGATGTTGATTGG	266	60.4
FP206	comp162751_c0_seq1	(CAA)7	TCCTGCCCCAATTGTTAGTC	CAGCACAATAAGGGCGAAAT	172	60.0

FP207	comp162770_c0_seq1	(TG)8tatc(TG)6	GCCAATTGACTGCAACAACA	AATTCCATCCTACCCAACCC	245	60.3
FP208	comp163104_c0_seq1	(TA)9---(TA)8	TGGGAACAAATTAGCGGTTC	AGTTGAGGAGGGTTTGGTGA	150	59.7
FP209	comp165582_c0_seq1	(TG)6cgtgtgtgt(GA)6	TCTGGCCCTAGAACTCATGG	TTTTTCTTTCCATTTTTAGTCCC	172	59.0
FP210	comp165803_c0_seq1	(TG)7(CA)6	GATGATTGGGATTGGTCGAG	TCAGGGCATAACTAACGGCT	133	60.0
FP211	comp167371_c0_seq1	(AC)10	TGGGGAAGTGACCAGAAAAG	CATAAGACCATGCCTGCACA	133	60.4
FP212	comp168550_c0_seq1	(TA)7---(AT)7	TGGATATTCAAGGGATGGGA	TGCAAAATTGTATCCTTACCTGT	247	58.9
FP213	comp170622_c0_seq1	(TTTA)5	CCCCTTTTGTAGATGCCTCA	AATTGAGACAGGCGTTTTGC	128	60.2
FP214	comp17468_c0_seq1	(CTT)7	TGTTTGGTGTTGGAGCACAT	AATTTGTTGCAAAACAGGGC	132	60.0
FP215	comp175724_c0_seq1	(ACC)7	TCGCTTCTCCAATCTCGACT	GTCGATCGCAACCATTCTTT	104	60.1
FP216	comp177028_c0_seq1	(ACA)7	CAAAATCTTAGCCGGAGCAA	CCAACATCACAATCACGTTTC	145	59.6
FP217	comp178229_c0_seq1	(GA)6cgagag(GA)7	GGAGAGAGAGAGGGAGCGAT	CGGTTTCATATAAACTTTTCCCC	100	60.0
FP218	comp178349_c0_seq1	(ATTA)5	ACCTCGATCCCCCAATAATC	AATCCACACAGCTTTTTATCTGTT	121	59.2
FP219	comp178867_c0_seq1	(TG)6(CA)7	CAGGGCATAACTAACGGCTC	GATGATTGGGATTGGTCGAG	132	60.0

FP220	comp179111_c0_seq1	(GC)6---(TG)6	TGCCATTTAAGATCAATGGAAG	AAGTGGGCAGCTGAAAAAGA	148	59.5
FP221	comp179499_c0_seq1	(TA)8a(AT)8	GCTGGCAAGTGTGTAAGCAA	GCCATCCCTAATCGTGATTGT	208	60.6
FP222	comp179524_c0_seq1	(TATG)5	CGAAGACTGGGACTCGAAAG	CAGGTCAAATTTTATGACTAAATCCA	198	59.8
FP223	comp180062_c0_seq1	(TTTA)5	CAAAGCATTCAGGCAAATCA	CACATTTTGCCACGTACACA	120	59.4
FP224	comp181280_c0_seq1	(GA)7---(GA)8	GATCTGAGGGGTGAGGGAAC	CATTTTGGGCCCGTTATTTA	121	60.3
FP225	comp182622_c0_seq1	(AC)10	CACTTGGCACATATCGATCAC	TGCATCGTCCACTTACAACAA	137	59.6
FP226	comp183492_c0_seq1	(GA)10	TCACCATGAAGCTTGAAAAGAA	TAACCGTGTTGTCGTTGCTG	111	60.3
FP227	comp184_c0_seq1	(AATT)5	TGAGTCCGTGTCTTGTAGCG	CCAAAATGCTGTTTTCTTGGA	280	60.1
FP228	comp184131_c0_seq1	(CAT)7	ACCATTTTTACCGCCAATCA	CATTTTGATGGGGAAGAGGA	148	60.0
FP229	comp187366_c0_seq1	(CATG)5	TGGAACAGATGAATAGACAGCA	AAGGACATGATAACGCGGAC	235	59.2
FP230	comp189467_c0_seq1	(TTTA)5	TCCTCATTTGCTGTTGCTTG	TGCTCTTGTGGCAGTAATGG	207	59.9
FP231	comp190124_c0_seq1	(A)10t(AG)7	TCCTGTAAGGTTGTGCGTTG	GAAGTGGTCGGCAACTCAAT	143	59.9
FP232	comp194592_c0_seq1	(TATT)5	AATAGGAACGGTTCGGCTCT	CATGGCTTTAGAAAAAGGCG	105	60.0

FP233	comp196805_c0_seq1	(TTA)7	TCATGAAAAATTGTTCCCCC	CTTTACCACGGGATGCAAAT	168	59.7
FP234	comp21622_c0_seq1	(GAT)7	AAAGTAGTCCTGCAAAGCGG	AGGGAGCTTTGTGTTGGGTA	171	59.6
FP235	comp23369_c0_seq1	(CT)7---(TC)8	ACGACATCCTCTCCAAGGTC	GAACGAGAGAGTAAAGGAGAGAGA	115	58.4
FP236	comp32258_c0_seq1	(ATAA)5	TCCACAGTGTGCATCCAAGT	ACAGCTTCATTCCATCAGGG	213	60.1
FP237	comp3240_c0_seq1	(AT)10	AGATCTTGCTCCGGCACTTA	TTCGTTATGCCTTGCATTGA	150	60.1
FP238	comp32764_c0_seq1	(GA)6---(GA)6	GTGAGAGAGGGAGAGGGGAG	TCTCCCTTCCGATGAGCA	220	60.4
FP239	comp34642_c0_seq1	(ATAA)5	AATTTTGAACATTGTATCAGACCA	GTTGATCGTGGGGAAATCAT	175	58.6
FP240	comp35232_c0_seq1	(CG)6---(CG)6	TGCCCTAAATGTCATGGTGA	TTTAGGGAATACATTCGACAAGTG	230	59.7
FP241	comp36495_c0_seq1	(AAG)7	TTCTTTTTCTCTCCCTCTACAACC	TCTTCCGTCAGTCCTATGGC	236	59.8
FP242	comp39872_c0_seq1	(CA)7tggg(GA)8	GGCAATCTGAAGCACATCAA	CCCTGGGCATCATCTAGAAA	247	59.9
FP243	comp40763_c0_seq1	(TTC)7	TCTCGTTGGATTCCTTTTGG	CTTGATAATGGGGTCACGCT	144	60.0
FP244	comp42511_c0_seq1	(TC)6---(TC)7	CCTCTCCGATCCTCTTTCCT	GGAGGTGAAGAGGGAGGC	255	59.7
FP245	comp43171_c0_seq1	(TTC)7	GGAAGGTTTCCCTCCTTCTG	GCGTTGCTATCCAATGGTTT	132	60.0

FP246	comp43430_c0_seq2	(ATTG)5	CAATACCGGCTAGCACCAAG	GTCAATCAATCAATGGCACG	149	60.3
FP247	comp44581_c0_seq1	(CT)10	ATGGACTGTTGGGTGCTTCT	CCATTGCAGTCGAAGTCTCA	210	59.8
FP248	comp44632_c0_seq1	(CCG)7	ACAAATAGGCCCCAAATTCC	GAGGGAAAATGGTGAGCAAA	104	60.0
FP249	comp45052_c0_seq1	(CTT)7	TGTGGGTTCTATGCTTTCCC	CCCCTGCTTCATCTCCAATA	137	60.0
FP250	comp45191_c0_seq1	(ATG)7	TTGCCTTTATTGGAAGCCAC	TGCACTCACTACCACCAAGG	247	59.9
FP251	comp4661_c0_seq1	(GA)10	GGATCCAAGAACGCAAGAAG	TCTCTTTCTCCCCTACCCGT	163	59.9
FP252	comp46986_c0_seq1	(GA)7---(GA)6	GGAGGTGAAGAGGGAGGC	CCTCTCCGATCCTCTTTCCT	255	59.7
FP253	comp47844_c0_seq2	(AAT)7	ATAATTCCGGAGAGATCGGC	ACTTCTCCCACCCACCTTCT	226	60.2
FP254	comp47868_c0_seq1	(AGG)7	CATGTAGAACAATCGCCCCT	TTGCACTGCGGTGACATACT	226	60.1
FP255	comp48939_c0_seq1	(ATTT)5	AGGAAATCATTGGAGACCGA	AATATAGCCCCTCTCACCCC	149	59.4
FP256	comp49359_c0_seq1	(AT)10	CAATGGGTTCACTGTCGATG	ATTCCAACTTGCAGCCATGT	236	60.2
FP257	comp50436_c0_seq1	(CT)10	CAGCGCTCTTGCTCAGTAGA	ACTCAGGGTCATGAAAACGG	145	59.8
FP258	comp50467_c0_seq1	(TGA)7	CCCTTCGATGAAGTGATGGT	AAACCAAAACACAACCCCAA	174	60.0

FP259	comp50726_c0_seq1	(CT)8tt(CA)7	TCTCCAAAACCTTCTTCTCACA	CTCGAGGAGGAGAGATGGGT	124	59.9
FP260	comp51989_c0_seq1	(GGA)7	TTTGTGGGGAGAAAGGAGTG	AAAATTGCTTTTCAAGCGGA	248	60.0
FP261	comp54646_c0_seq1	(ACAA)5	ACAAAACACCCACACCCACT	AATCTCTGCATGTCCTTGCC	148	60.2
FP262	comp54661_c0_seq1	(CGA)7	CCACCACAAGTTCAAACCCT	GGTTTTCGAATCGTCATCGT	217	59.9
FP263	comp56963_c0_seq1	(AATA)5	TGCAGATACCACCCAAACCT	AGGAAAACGCATACACGCAT	196	60.5
FP264	comp58610_c0_seq1	(GA)6---(GA)6	GTTTAGATGCCGATGGGAGA	CTGTCGCCATCCTAATTTCC	211	59.8
FP265	comp65808_c0_seq1	(TC)6(AC)6	AGACTTTAATGAGTCTCGCAGG	CCTTATTTGGTCCCATCCCT	198	58.9
FP266	comp66052_c0_seq1	(TC)6(T)10	ACTGCTGTCAACGAGAGGGT	AGATGAGCAATGGAGGAACG	268	60.1
FP267	comp66421_c0_seq1	(AC)8(GC)7	ACTCTCGTCATCCCGTCATC	GTTAGTGTGTGTGCACGGGT	121	59.8
FP268	comp68_c0_seq1	(AG)7ggg(GA)6	GTTAGGCCTAGGGATTTGGG	GCTTCGTTCCCTCTCTTCCT	217	59.9
FP269	comp68178_c0_seq1	(CT)11g(TA)7	TCAGTTTTAAAGCTTGCTGTGG	CTACCGCTTTGAGGAGTTCG	207	59.8
FP270	comp68477_c0_seq1	(AG)10	TGGAGCTGCCAGGATTTTTA	CCTAACCCTAGCTTCCCCAA	280	60.6
FP271	comp68831_c0_seq1	(GA)10	ACTCAGGGTCATGAAAACGG	CAGCGCTCTTGCTCAGTAGA	145	59.8

FP272	comp70280_c0_seq1	(GTG)7	TCCGTATCTTGGGATTAAGGTTT	TCGCTCGACCTTGTATTTCC	166	60.1
FP273	comp71140_c0_seq1	(TCG)7	ACCGGTGCTTGCTTCTTCT	GAAACCCAAAAACCCTAGCC	109	59.9
FP274	comp72567_c0_seq1	(AGA)7	AAATTTGACCTGCCGAAAAA	TCCTTCTGCCCAATTCATTC	168	59.8
FP275	comp734_c0_seq1	(TTA)7	AGGCGATGTCTCCATGAATC	GTTGATCGCTCTATCGACCC	165	59.9
FP276	comp74104_c0_seq1	(TCG)7	GTTGTCGAGGTCCCAGTCAT	CCACCACAAGTTCAAACCCT	163	59.9
FP277	comp75497_c0_seq2	(TTAT)5	GTTGAACCAGCAGCACTTCA	ATGGTGACATCTCAACGCAA	269	60.1
FP278	comp76274_c0_seq1	(TTC)7	AAGCTCGCATAGGTCTCCAA	CGTTAGAATTTGGGGTCGAA	185	60.0
FP279	comp76820_c0_seq1	(TTTC)5	ATGTCAAACGGACAATGCAG	ATCACCAAACCATCCCAAAA	249	59.8
FP280	comp76887_c0_seq1	(AATA)5	TGAACAGTGAAACTCGGCAG	TGTGGTGGAAATTTTCTTCATT	179	59.2
FP281	comp77248_c0_seq1	(GT)10	TACATCCATGGGGGCATATT	ATGAGGTGTTTGGGCTTTTG	107	59.9
FP282	comp77447_c0_seq1	(CA)10	AAGTGGCCGAACCCTTACTT	TGCCCTGAGTGACTTACACG	238	59.9
FP283	comp79840_c0_seq1	(CA)10	GTCACCTTTGTACCCTGGGA	GGCTTTGATCCTATTGTGGC	185	59.7
FP284	comp7989_c0_seq1	(TGA)7	CAAAGGCAAGGGGTTTAAGA	GCCTAACAAACAGGCCCTAA	248	59.2

FP285	comp80689_c0_seq1	(TC)6---(CT)6	TCCATCTCCACACCTTCCTC	GGTGATAGAGGGAAATGCGA	271	60.0
FP286	comp81189_c0_seq1	(TCA)7	ATGCATTTGCAAGCTCTCCT	GTGATCCTCTGCCGAATCAT	141	60.0
FP287	comp81440_c0_seq1	(TG)11	GATTCCCATTTTCCTCAGCA	CACTCACACAAACACACCCA	117	59.5
FP288	comp81489_c0_seq1	(TTAT)5	TTTTCTTCATTACTGACCTGTTTGA	TAACATTACGCCTGGCAACA	214	59.7
FP289	comp81981_c0_seq1	(CCG)7	TTGGGTAACCCTGAACCAAG	GGCCAAATCAAGAAACCTCA	148	59.9
FP290	comp82174_c0_seq1	(TCT)7	TCTTCCGTCAGTCCTATGGC	GTTTCGGTACGAGACCTCCA	237	60.2
FP291	comp82510_c0_seq1	(TTAT)5	AAGCCTCGTGAAACTCTCCA	AACTGCTTCCGGATAAATGC	200	59.6
FP292	comp82743_c0_seq2	(AATA)5	GATGCAAGAAGGGTTCCAAA	TTGCATTTTAACAGCGCAAG	114	60.0
FP293	comp82850_c0_seq1	(ACA)7	GTCCAAAACCGACGAAAAGA	TGCATCCACTTTCTCAGTGC	269	60.0
FP294	comp84738_c0_seq1	(ACC)7	TCTCCAAGAGGCCAACTGAT	GACCTCCCTCCACAGATTCA	264	59.9
FP295	comp84788_c0_seq1	(GAA)7	CGTTAGAATTTGGGGTCGAA	TCTTGAGAGAGGAAGCTCGC	197	60.0
FP296	comp84924_c0_seq1	(AG)8acg(GA)6	TGCTCTCCTCTTTCTCTAACACAA	GCAACGGTCTTGAAGTGGAT	250	59.9
FP297	comp87358_c0_seq1	(AG)7---(AG)7	TGGGTTGGAGGTGGAGTTAG	ATCACCGACGACATCCTCTC	115	60.0

FP298	comp87487_c0_seq1	(GGC)7	ATAACTGAAAAGGAGCGGGG	CCAAGCAGCAACACAATCAC	226	60.4
FP299	comp88505_c0_seq2	(CAC)7	AGCAATGCAAAGCTGATCCT	ATCATCCGAGTCGGTAGTGG	240	60.0
FP300	comp88619_c0_seq1	(TC)10	AGGGCTCTTCTGACGATGAA	TTTTCCTCTCTTCCCAATGC	245	59.6
FP301	comp88838_c0_seq1	(CA)10	CTAAACAGACCCCCAACCAA	TATCCCTGATGAATCCGCTC	218	59.9
FP302	comp89275_c0_seq2	(CT)6cgctct(CG)6	CCCGGGTATTCAACGATAAG	GCAGATGTTGAGGAAGAGGC	194	59.6
FP303	comp89278_c0_seq1	(TCA)7	CCCAAACATCACCAATTCAA	CCAAGGAGTACGAGGACCAA	128	59.7
FP304	comp89620_c0_seq2	(TC)10	GGGAAAAGAAAACAGAAGGGA	TGGTGGTGGAAAAATGGAAT	189	59.8
FP305	comp90811_c0_seq1	(CAA)7	CACCATGCCAACCTTCTTCT	CCTGCTGAGATTTTGCCTTC	167	60.0
FP306	comp90885_c0_seq1	(GGA)7	GTGGTTGGGGTTATGGTACG	AACCAAGTAGTGAAGGCCCC	152	60.2
FP307	comp91361_c0_seq1	(TTAA)5	CCCTGCTTCTTTCAATCCTG	AGGGGTGCTTGGTTACTTTG	164	59.5
FP308	comp91736_c0_seq1	(AAG)7	TTTCCAAGAGCCTGACGAGT	CTCCCCTCCATCATCTTTGA	214	60.0
FP309	comp92146_c0_seq2	(TGG)7	AGAGGACCTTGAGATTGGCA	CTCGAACAGCTCCTCCAAAC	256	59.9
FP310	comp92593_c0_seq2	(AG)12	GGAAGGGAAATGGGGATAAA	CCCAATGCCAAGAAAGTTGT	167	60.0

FP311	comp92627_c0_seq1	(CA)6--(TG)7	ATAACATGTCCATGCGTTCG	TCCTGCCTTCTTCCAGCTTA	157	59.8
FP312	comp92809_c0_seq1	(GTTT)6	GGTGTCAACAACACACCGTC	TAACCATTCTGCCACCACAA	200	59.9
FP313	comp93149_c0_seq1	(CAT)8	TGAATCCCTTCTTCCATCTCC	TTGAAGGAAGAATATGGCGG	174	60.2
FP314	comp93595_c0_seq1	(GA)10	TTGACAAGTGTCGCTGTGCT	CAGGATAATCGCGTCACTAGA	203	59.3
FP315	comp95251_c0_seq1	(GGA)7	GCAGTGATTCTCCACCCACT	TCCCTCCTTAGATTCCCCAT	277	59.9
FP316	comp95731_c0_seq1	(CATT)5	CCACTTCTTCCACCAACACC	GTTTTGCAACGAAGACGACA	267	60.1
FP317	comp95886_c0_seq2	(GA)6gtg(GA)7	TGAATGCATGGGTGAAAAGA	ACCCTCCATCCCCTTACTGT	161	59.9
FP318	comp96361_c0_seq1	(GGA)7	GATGTTGGTGGTGGAGAACA	ACCCCTTTCCTTGCCTTCTA	269	59.7
FP319	comp96452_c0_seq3	(GTTT)5	CGGTAGATGGGGCATTAAGA	AATTCCCGAACACTGGTGAG	150	59.9
FP320	comp97022_c0_seq2	(AC)11	TACCAACATTTCGCCTACCC	TTTGCAAATCATCAAGTTCAGTG	136	60.0
FP321	comp97533_c0_seq3	(AC)6--(AC)8	TTTGCCATCATCCAGATTCTC	ATGTTTAAAATGATACGGTTTGATAA	218	58.7
FP322	comp97645_c0_seq1	(AAT)7	ACCAAGATGGTGGCAAAAAT	CCTTCAAGCCCTCATCTTCA	235	59.8
FP323	comp97722_c0_seq1	(GAA)7	GCTTTTTCTGTGCTCGGTGT	AAGTGACACGGGCATTTTTC	172	60.2

FP324	comp97815_c0_seq1	(GA)10	CACAAATCATTCCCCAATCC	AGTCGACAACCACGAGCTTT	195	60.0
FP325	comp97941_c0_seq1	(CT)7at(AC)9	CACCAAGATGGCTGTGCTTA	CCATTTCTGTTGCTCCTTCC	237	59.8
FP326	comp98049_c0_seq1	(AATT)6	ACATGGCCGACTAGAAATCG	CGTCTGTCAACAGTTCCCAA	224	59.9
FP327	comp98476_c0_seq1	(AC)7---(TG)6	TCCTGCCTTCTTCCAGCTTA	AAGAAATAACATGTCCATGCGTT	174	59.9
FP328	comp98660_c0_seq1	(AG)10	TGGAGTGGGGTTTCTCTCAG	TCGCTTTGGGTAAATCAAGC	277	60.2
FP329	comp98837_c0_seq1	(AT)10	CAAATGTCCGAGAGTCCAGA	CACTCGCACGAGGAAACATA	265	59.3
FP330	comp98945_c0_seq1	(GCT)5--(GTT)7	TTCGGTAGCGAGTCTGGTTT	TTTCAATGAATCGCCAAACA	273	60.0
FP331	comp98981_c1_seq2	(AG)8---(GA)6	GACATCACCGTCGACATCAC	CCTTCCGCTAATTCCGGC	172	61.4
FP332	comp98986_c0_seq1	(AAC)7--(AGC)5	TTTCAATGAATCGCCAAACA	TTCGGTAGCGAGTCTGGTTT	273	60.0
FP333	comp99022_c0_seq1	(ATA)7	TGGGTACCGATTGGAAATGT	GGTCAGACCAGATTCCCTGA	245	60.0
FP334	comp99195_c0_seq2	(GGC)7	TCCTCATCCACCACTCTTCC	AACACCCAACTGTCAAAGCC	216	60.0
FP335	comp99659_c0_seq1	(TCA)7	CCATCTCTCAGCAACCATCA	TTTCAATGGCAGCCTCTCTT	228	59.9

Table S5. The 104 accessions of Hippeastrum used in present study for transcriptome sequencing and SSR markers development.

Cultivars/hybrids	Origins	Spathe Color	Type
Game	Netherlands	pink	single petal
Ludwig Dazzler	Netherlands	white	single petal
Splash	Netherlands	red and white complex	double petal
Hercules	Netherlands	red	single petal
Faro	Netherlands	pink	single petal
Pink surprise	Netherlands	red	single petal
Mambo	Netherlands	red and white complex	single petal
Magic Green	Netherlands	red and white complex	single petal
Matterhorn	Netherlands	white	single petal
Apple Blossom	Netherlands	pink	double petal
Red Peacock	Netherlands	red and white complex	double petal
Amorice	Netherlands	red and white complex	single petal
Exotic star	Netherlands	pink	single petal

Exotic peacock	Netherlands	red and white complex	double petal
Minerva	Netherlands	red and white complex	single petal
Gervase	Netherlands	pink and red complex	single petal
Bellisimo	South Africa	pink or red	single petal
Joker	South Africa	red and white complex	single petal
Benito	Netherlands	red	double petal
babydoll	South Africa	pale green	single petal
Mont Blanc	Netherlands	white	single petal
Ballerina	South Africa	red	double petal
Popov	Netherlands	red and white complex	single petal
Charisma	Netherlands	red	single petal
Sweet Nymph	Netherlands	pink	double petal
Hot lips	South Africa	pink	single petal
Double Dream	Netherlands	pink	double petal
Hollywddd	South Africa	pink	single petal
Marilyn	Netherlands	white	double petal

Santiago	Netherlands	red and white complex	single petal
Misty	Netherlands	pink	single petal
Flanmingo	Netherlands	red and white complex	single petal
Cherry Nymph	Netherlands	red	double petal
Royal Velvet	Netherlands	red	single petal
Arctic Nymph	Netherlands	white	double petal
Susan	Netherlands	pink	single petal
Baby Star	Netherlands	red and white complex	single petal
Double delight	Netherlands	red	double petal
Ice Queen	Netherlands	white	double petal
Vegas	South Africa	red and white complex	double petal
Harlequin	South Africa	white	double petal
Chico	Netherlands	pink	double petal
La Paz	Netherlands	red	single petal
Rock roll	South Africa	red	single petal
Razzledazzle	South Africa	red and white complex	single petal

Candy Floss	South Africa	pink	single petal
Showmaster	Netherlands	red	single petal
Temptation	Netherlands	red and pink complex	single petal
Purple Rain	Netherlands	pink	single petal
Vera	Netherlands	pink	single petal
Adele	Netherlands	red	single petal
Exotica	Netherlands	Orange	single petal
Double Record	Netherlands	white and pink complex	double petal
Exotic Nymph	Netherlands	white and pink complex	double petal
Fanrare	South Africa	red	single petal
Pretty Nymph	Netherlands	white and pink complex	double petal
Red Nymph	Netherlands	red	double petal
Jewel	Netherlands	white	double petal
Nymph	Netherlands	white and red complex	double petal
Ragtime	South Africa	red	double petal
Stardust	Netherlands	red	single petal

Blushing Bride	South Africa	pink	double petal
Lemon Sorbet	South Africa	green	single petal
Zombie	South Africa	white and pink complex	double petal
Benfica	Netherlands	red	single petal
Brazza	Netherlands	red	single petal
First Love	South Africa	white and pink complex	double petal
Rozetta	South Africa	pink	double petal
Sunny Nymph	Netherlands	red	double petal
Double king	Netherlands	red	double petal
Pasadena	Netherlands	red	double petal
Elvas	Netherlands	white and pink complex	double petal
Papillo	Netherlands	red and green complex	single petal
Neon Eon	Netherlands	pink	single petal
Amafi	South Africa	pink	single petal
Rilona	Netherlands	red	single petal
Red Lion	Netherlands	red	single petal

Lady Jane	Netherlands	red	double petal
Green Goddes	Netherlands	white	single petal
Fairytale	Netherlands	red and white complex	single petal
Dancing Queen	Netherlands	red and white complex	double petal
Carmen	Netherlands	red	single petal
Aphrodite	Netherlands	white and pink complex	double petal
Amigo	Netherlands	red	single petal
Ambiance	Netherlands	red and white complex	single petal
13—40	Netherlands	red and white complex	single petal
13—41	Netherlands	pink	single petal
Ferrari	Netherlands	red	single petal
Intokasi	Netherlands	white	single petal
13—46	Netherlands	red and white complex	single petal
13—47	Netherlands	red	single petal
Carina	Netherlands	red	single petal
10—6	Netherlands	red and white complex	double petal

Desire	Netherlands	orange	single petal
Orange	Netherlands	red	double petal
10—32	Netherlands	red and white complex	double petal
Red Knight	Netherlands	red	single petal
Lemon	Netherlands	lemon	single petal
Alfresco	Netherlands	white	double petal
13—55	Netherlands	red and white complex	double petal
Flaming Peacock	Netherlands	red and white complex	double petal
10—04	Netherlands	red and white complex	double petal
Amorice	Netherlands	red and white complex	single petal
Bolero	Netherlands	red	single petal
